# Making sense out of uncertainty: cognitive strategies in the child custody decision-making process

**DOI:** 10.3389/fpsyg.2024.1387549

**Published:** 2024-07-15

**Authors:** Josimar Antônio de Alcântara Mendes, Thomas C. Ormerod

**Affiliations:** ^1^Department of Computer Science, Responsible Technology Institute, University of Oxford, Oxford, United Kingdom; ^2^School of Psychology, University of Sussex, Brighton, United Kingdom

**Keywords:** child custody, naturalistic decision-making, divorce, uncertainty, cognitive strategies

## Abstract

Child custody cases post-parental separation entail inherent complexities and uncertainties for legal experts and decision-makers, and are influenced by context factors. This study sheds light on how legal actors (i.e., judges, prosecutors, lawyers, psychologists, and social workers) navigate the uncertainties that arise in such context and, therefore, make their decisions. Based on a reflexive thematic analysis involving 73 participants from Brazil and England, this study reveals cognitive strategies employed by legal actors to comprehend uncertainty and operate in the decision-making context. These strategies encompass heuristics (i.e., selection, evaluation, degrees of freedom, and outsourcing decisions/ resolution) and metacognitive strategies (custodial arrangements, professional practices and ‘best interests of the child’ speech). These results provide a window into the decision-making processes in child custody cases; they offer a comprehensive understanding of the multifaceted sensemaking strategies employed by legal professionals. The results carry substantial implications for informing and improving legal practice in handling complex child custody situations. Furthermore, this study charts new paths for future research by highlighting potential avenues for refining and advancing the strategies employed by legal experts in these cases, especially considering the child’s best interests.

## 1 Introduction

After a divorce^1^, most separated parents manage to reach a settlement regarding child custody^2^ and/or contacts/access with their children. However, some go to the family court to seek a decision regarding these matters – this occurs in about 5% of divorce cases (Kelly, 2002, 2007; Wallace and Koerner, 2003; Baker, 2011). Despite being a small number of cases, judicial disputes are challenging to professionals involved in child custody decision-making. They tend to require considerable resources, especially when parents apply to court multiple times, even when the child reaches legal adulthood (Cano et al., 2009; Antunes et al., 2010; Rosmaninho, 2010; Juras and Costa, 2011; Hashemi and Homayuni, 2017; Mendes and Bucher-Maluschke, 2017a).

One might wonder what makes that 5% of cases evolve into complicated situations that require the involvement of legal experts to make a decision? The answer is threefold: (1) families face developmental struggles after parental separation (e.g., being unable to differentiate issues of the conjugal relationship from the parental/coparental one), which makes it difficult for the family to overcome the crisis moment they face during divorce (Mendes and Bucher-Maluschke, 2017a) – these issues structure most of the uncertainty in child custody cases after parental (Mendes and Ormerod, 2023); (2) environmental and organizational issues that impact the decision-making process (e.g., professionals’ workload) but also the families’ coping strategies (Mendes and Ormerod, 2023); and (3) how legal experts respond to the interaction between family issues and environmental/organizational issues (e.g., strategies they use to navigate the decision-making environment and make sense of the information they face). Mendes and Ormerod (2023) have shown these factors play a significant role in child custody cases. However, these factors tend to be underestimated and dismissed by legal professionals, which increases uncertainty (Mendes, 2022).

These three domains can be viewed as sources of uncertainty driven by ‘contextual factors’. These factors encompass elements and dynamics related to individual, system, and organizational issues that influence decision-making by introducing uncertainty (Mendes and Ormerod, 2023). Arguably, every decision-making process carried out in a natural setting is surrounded by uncertainty (Klein et al., 1993; Lipshitz, 1993a,b; Lipshitz and Strauss, 1997; Lipshitz et al., 2001). Hence, addressing contextual factors is important as they structure the uncertainty in child custody cases – which can blur the problem’s perception or its possible solutions (Lipshitz, 1993b; Lipshitz and Strauss, 1997; Mendes and Ormerod, 2023).

In this study, we considered uncertainty as encompassed by doubts generated by the perception of a problem and its structure and shape in search of a solution (Lipshitz, 1993a; Lipshitz and Strauss, 1997). In child custody cases, contextual factors prompted by the family typically relate to psychosocial (e.g., developmental struggles) rather than legal issues (e.g., contacts with and access to the child after the divorce), which can increase the stress that impacts legal professionals’ performance and even their mental health (Mendes and Bucher-Maluschke, 2017a,b). Mendes and Ormerod (2023) conducted a cross-cultural qualitative study between Brazil and England, interviewing legal professionals (judges, prosecutors, lawyers, social workers, psychologists). They identified three domains of contextual factors that impact the child custody decision-making process.

As shown in Table 1, those domains are: (1) *family*: issues concerning the family dynamics and its developmental struggles. For instance, issues may relate to the family life cycle, parenting, co-parenting, and coping strategies after divorce; (2) *family court*: issues concerning organizational and legal matters. For instance, issues may relate to law procedures, staff numbers, workload, and how the court addresses the child during court proceedings; and (3) *legal-psychosocial*: issues concerning the psychosocial evaluation process that informs the decision-making process – conducted by either social workers or psychologists that will look at the child’s general needs and welfare.

**Table 1:** Themes by context factor domain.

**Context factor domain**	**Theme**
*Family*	Theme CT1: Parental Separation: Crisis and Family Life Cycle CT1.1. *Dysfunctionally coping with divorce: family crisis* CT1.2. *Misunderstanding and pathologization of family interactions and coping strategies in the context of custody dispute: perspectives on parental alienation* CT1.3. *Parental separation as part of the family life cycle* Theme CT2: Hindering the Best Interests of the Child CT2.1. *Conjugality* vs. *Parenthood* CT2.2. *Detaching from the child and attaching to the litigation* CT2.3. *Lack of parenting skills* CT2.4. *“No ‘child maintenance’, no contact with the child”* CT2.5. *Misunderstanding joint custody* CT2.6. *Involving the child in parental conflict* Theme CT4: Applying the Best Interests of the Child CT4.2 *Idiosyncrasy* Theme CT5: Making the Decision-making Process Harder CT5.1. *Misconduct, maltreatment and abuse allegations* Theme CT7: Making a Custodial Arrangement Involving Adolescents CT7.2. *“They can play the game too”: getting into the litigating parents’ dynamic*
*Family Court*	Theme CT3: The Judiciary’s Constraints and Practices CT3.1. *“The Law is powerless”: legal and epistemological limitations of law* Theme CT4: Applying the Principle of the Best Interests of the Child CT4.1. *Indeterminacy* Theme CT5: Making the Decision-making Process harder CT5.2. *Tied parents: “I cannot pick one”* Theme CT7: Making a Custodial Arrangement Involving Adolescents CT7.1. “*It’s quite impossible to go against their will”*
*Legal-psychosocial*	Theme CT3: The Judiciary’s Constraints and Practices CT3.3. *Between fear and bravery: the psychologists’ practice in Brazil* CT3.4. *An advocate in intractable cases: the psychologists’ practice in England* Theme CT6: Assessing the Best Interests of the Child in Child Custody Cases: Evaluation Services CT6.1. ‘*Psychosocial study’: the Brazilian model* CT6.2. *Children and Family Court Advisory and Support Service – CAFCASS’: the English model*

Source: Mendes and Ormerod (2023).

Decision-making in child custody cases is also influenced by the type of legal system in a country. In the context of this study, England operates under a Common Law legal system, rooted in ‘customary law’ derived from tribunals and prior decisions. Conversely, Brazil follows a Civil Law legal system, grounded in ‘positive law’ emanating from enacted statutes – for further discussions see Mendes and Ormerod (2021). An additional legal factor impacting the decision-making process is the best interests of the child principle (BIC), which holds significant importance in the child custody decision-making process in the Western world (Mendes, 2024). However, definition and operationalization of BIC after parental separation is quite challenging. This is due not only to the lack of training and/or education among legal actors (judges, prosecutors, lawyers, psychologists, social workers) regarding this principle (Mendes et al., in press), but also because the context of child custody decision-making after parental separation is inherently complex and marked by uncertainties (Greene et al., 2012).

The BIC principle varies based on each country’s culture and values (Mendes and Ormerod, 2019). In child custody cases, the BIC dictates that whatever is best for the child’s developmental needs and general welfare should not only be considered but prioritized above any other concern, issue, or demand from adults or institutions (Mendes and Ormerod, 2021, 2023; Mendes, 2022; Mendes et al., in press). However, some legal professionals perceive the BIC as a vague and under-determined principle, introducing more uncertainty into the decision-making process (Mendes and Ormerod, 2021; Mendes, 2022) – these claims generally come from professionals who do not have complete knowledge of the BIC and its origins (Mendes, 2024).

The final decision is for the judge to make by weighing the evidence presented by both parents and ultimately determining a resolution for the case and the child’s best interests (Mendes and Ormerod, 2021; Mendes, 2024). However, the child custody decision-making process is framed by inputs coming from legal professionals with different roles, tasks, and objectives: prosecutors, lawyers, psychologists, and social workers (Mendes, 2022). In Brazil, but not in England, prosecutors are involved in child custody cases. Their role is to secure children’s rights and general welfare by supervising the decision-making process according to legal standards – see Mendes and Ormerod (2021). Lawyers become involved in child custody cases to advocate for each parent’s interests, and in some instances, these interests may not align with those of the children. This discrepancy often arises when parents hold opposing views and advocate different perspectives on what is in the child’s best interests. Psychologists and social workers are typically involved when additional evaluation and information are required to assess the child’s biopsychosocial and emotional well-being (Mendes and Ormerod, 2021). In theory, the role of psychologists and social workers is to prioritize and focus on the child’s best interests during the decision-making process. However, in both Brazil and England, there are some critiques regarding their evaluations, which arguably tend to be non-protocol and non-evidence-based (Mendes and Ormerod, 2023). All these diverse roles, tasks, and objectives contribute to uncertainty, making it challenging for legal professionals to make sense of ‘what is going on’ in the case. As a result, it becomes more difficult for them to make efficient decisions that would effectively safeguard the child’s best interests.

In addition to the interaction and contributions from various stakeholders, the complexity of child custody cases is compounded by the quality of available information, particularly at the case’s outset. This information often tends to be incomplete, ambiguous, and contradictory. In certain instances, the severity of allegations, such as domestic violence, psychological violence and sexual abuse, proves challenging to investigate (Meier, 2017; Archer-Kuhn, 2018). This ambiguity leaves decision-makers uncertain about the actual risks and potential harm to the child’s best interests. These challenges intensify the pressure on the decision-making process, causing decision-makers to be apprehensive about the outcomes and their potential consequences.

These contextual, organizational, and legal issues appear to make the child custody scenario fit within a Naturalistic Decision-making (NDM) research framework. Considering the assertions made by Orasanu and Connolly (1993) and Mendes and Ormerod (2023), we see that child custody cases present: (1) *ill-structured problems* (non-organized; poorly presented; with contradictory allegations made by parents); (2) *uncertain dynamic environments* (complex and constantly changing due to the family’s developmental adaptations after divorce, which can display an erratic, confusing, dysfunctional, non-assertive, or disorganized picture); (3) *shifting, ill-defined, or competing goals* (multiple purposes that vary from case to case and involve issues regarding the child’s welfare, rights, and best interests, solving a legal issue, resolving parental conflict, and finding a solution that avoids future court applications); (4) *time restrictions* (time pressure that can lead to burnout and rationalization as a psychological defense mechanism among legal professionals) (Mendes and Bucher-Maluschke, 2017b); (5) *high stakes* (the child’s best interests); (6) *multiple players* (different legal professionals involved in the decision-making process, which can create discrepancies in understandings of the problem and the strategies available to solve it or even on what is a priority in a given case); (7) *action/feedback loops* (individual and cooperative performed by legal professionals throughout a case, such as applications made by lawyers on behalf of parents, evaluations carried out by social workers and psychologists, and the court’s intermediate decisions and actions); and (8) *organizational goals and norms* (judiciary’s and court’s values and goals; legal rules, guidelines, and standard procedures related to the hearings, mediation, or evaluation processes).

Scholars have extensively explored factors influencing the child custody decision-making process, looking at perspectives and values of lawyers and psychologists (O’Neill et al., 2018), the BIC (Eekelaar, 2015), family dynamics impacting judges’ understanding throughout the case (Wallace and Koerner, 2003), judges’ attitudes (Stamps et al., 1996), and standard evaluation procedures (Goldstein, 2016). However, there has been a notable neglect of the decision-making process itself, its characteristics, and steps, especially under an NDM approach.

NDM models, such as those proposed by Klein et al. (1993), tend to highlight decision-makers rather than the broader context of actors (e.g., parents, children, etc.) as crucial players in the decision-making process. In child custody cases, legal professionals and the family (including parents and children) are significant players who can directly and indirectly influence how legal professionals understand the case’s complexity and navigate the decision-making process (Mendes and Ormerod, 2023). Moreover, the limited NDM studies addressing child custody cases do not adopt a radical NDM approach (Taylor, 2007) or merely mention NDM in passing (Horta, 2019; Damman et al., 2020).

Given the lack of prior research on the child custody decision-making process under an NDM approach, this study aims to explore the cognitive strategies legal professionals employ to cope with the uncertainty prompted by contextual factors in both Brazil and England. The decision-making process in child custody cases is inherently complex, influenced by a myriad of factors, including cultural, legal, and organizational dynamics. Brazil and England present contrasting legal frameworks and cultural contexts, which significantly shape how legal professionals approach and navigate child custody cases. This cross-cultural study between these two countries offers a unique opportunity to explore the nuances of decision-making under NDM.

The contrasting approaches to child custody in Brazil and England are relevant for an NDM approach because they reflect distinct cultural and systemic differences that can naturally influence decision-making. For instance, in Brazil, child custody, referred to as ‘*guarda*’, includes sole and joint custody, with a legal preference for joint custody unless one parent opts out. This system often leads to prolonged litigation, intertwining custody decisions with child maintenance, which is strictly enforced by the courts. The Brazilian legal system emphasizes formal procedures and judicial decisions, which can extend the decision-making process and add layers of complexity. Conversely, England uses ‘child arrangements orders’ that do not employ the term ‘custody’ but focus on flexible arrangements tailored to each child’s needs and best interests. The English system encourages non-judicial resolutions and parental agreements, promoting the concept of parental responsibility over parental power (like in Brazil). The child’s welfare is the paramount consideration, guided by a comprehensive welfare checklist and the possible involvement of guardians *ad litem* in complex cases. The English system emphasizes flexibility and the resolution of disputes outside the courtroom, aiming to minimize conflict and prioritize the child’s well-being. For further discussions comparing child custody cases in Brazil and England, please refer to Mendes and Ormerod (2021).

By examining these differences through the lens of NDM, this study highlights how the unique contexts of child custody in Brazil and England shape the decision-making processes of legal professionals. Understanding these contextual influences can provide insights into improving decision-making practices and ultimately enhancing the welfare of children involved in custody disputes.

## 2 Materials and methods

This paper presents results derived from an extensive transcultural study conducted between Brazil and England. The main questions posed by the present exploratory and qualitative study were: (1) how the decision-making process is structured in terms of its context dynamics and constraints? (2) what is the role of legal actors in the decision-making process? These questions and their inherent objectives significantly shaped our selection of instruments, procedures, and participant recruitment methods.

### 2.1 Instruments, participants and procedures

This study utilized qualitative semi-structured interviews incorporating both open-ended and close-ended questions (refer to Supplementary material 1). The interviews involved 73 legal professionals, including prosecutors, judges, lawyers, psychologists, and social workers, with 48 participants from Brazil and 25 from England. Among the participants, 64% were female. The mean years of professional experience were 14 (SD = 9.7) in Brazil and 16.5 (SD = 8.9) in England. Brazilian participants were from Brasília, Porto Alegre, and São Paulo, while English participants were from various locations across the country. For a detailed description of the recruitment process, please refer to Mendes and Ormerod (2023). The interviews ranged in duration from 35 to 90 min, with an average of 5,478 words per transcript, varying from 1,440 to 11,552 words. Participants’ demographics are provided in Supplementary material 2.

Informed consent was obtained from all participants, and interviews were conducted either in person, via Skype, or by telephone in both countries, and recorded with a Sony ICDBX140 Digital Voice Recorder. This study and its materials (e.g., information sheet and consent form) were approved by the University of Sussex’s Social Sciences & Arts Research Ethics Committee under the Certificate of Approval number ER/JA454/2.

### 2.2 Data analysis

The data analysis process employed a ‘Reflexive Thematic Analysis’ (RTA) following the methodology outlined by Braun and Clarke (2022, 2023). RTA emphasizes and acknowledges the active and subjective role of researchers throughout the entire process, from data collection to analysis. This approach also incorporated insights and reflections from Nowell et al. (2017), Braun et al. (2019), and Mendes and Ormerod (2023), recognizing the significant role of the researcher’s critical perspective not only on the subject under study but also on the surrounding socio-cultural context – a comprehensive overview of the data analysis assumptions and epistemological discussions can be found in Mendes and Ormerod (2023).

**Figure 1:**
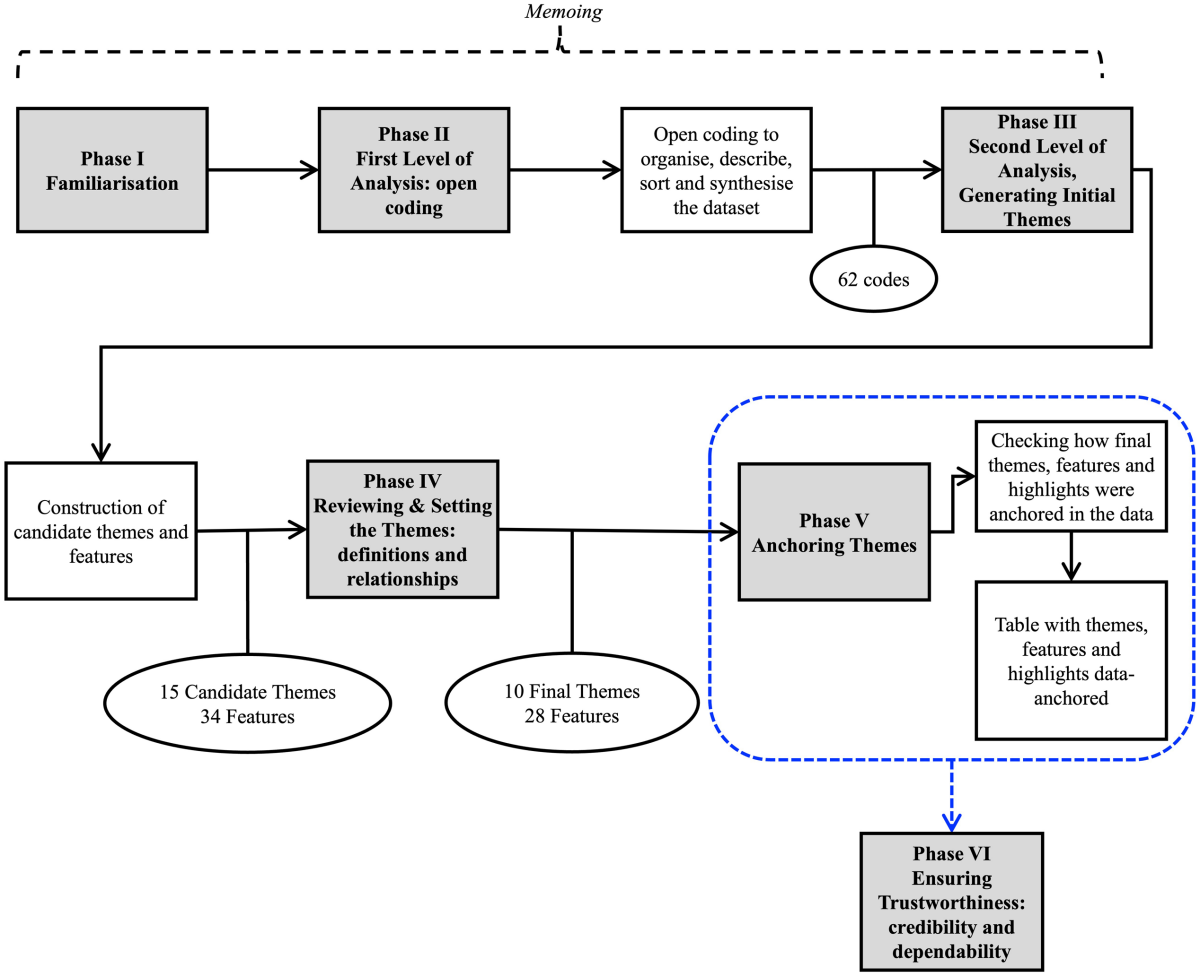
Data analysis process.

As seen in Figure 1, this RTA had six phases: Phase I – *Familiarization*: the first author read the entire dataset before initiating the coding process. The goal was to establish a close connection with the data, capturing initial patterns and common traits within the dataset. A memoing tool was utilized in this phase and throughout the entire analysis to document any emerging ideas, insights, and interpretations; Phase II – *First Level of Analysis (open coding)*: drawing inspiration from the concepts of ‘open coding’ as proposed by Urquhart (2013) and ‘initial coding’ by Charmaz (2014), the first author concentrated on organizing, describing, sorting, and synthesizing the data in an open manner aligned with the research questions. This phase was facilitated by the qualitative data analysis software NVivo 10 for Mac OS, resulting in the generation of 61 codes (refer to Supplementary material 3); Phase III – *Second Level of Analysis, Generating Initial Themes*: analyzing and integrating all the codes generated in the last phase to construct ‘candidate’ or intermediary themes. This phase generated 15 candidate themes and 34 features, see Supplementary material 4; Phase IV – *Reviewing and Setting the Themes, Definitions, and Relationships*: analyzing and improving candidate themes and features looking for more meaningful themes. This phase generated 10 final themes and 28 features; Phase V – *Anchoring*: illustrating how participants contributed to each theme/feature serves as a tool to enhance the dependability of the results. It is crucial to note that this approach should not be perceived as a quantitative measure where the significance of a theme/feature is determined by the number of supporters (participants) pointing towards it. For further details, please refer to Supplementary material 5; and Phase VI – *Ensuring Trustworthiness: Credibility and Dependability*: peer review and debriefing – four expert practitioners and academics, possessing expertise in child custody cases and/or qualitative research, thoroughly reviewed the data analysis process and the themes generated in this study. The first author conducted and documented a “Reflexivity,” a process of self-reflection used to increase awareness of the researcher’s actions, feelings, and perceptions (Anderson, 2008; Hughes, 2014). This process enables the researcher to recognize how their personal history, conceptions, and values influence the phenomena under study and to examine how their subjective role might introduce biases into the analysis. It serves as an alternative to assuming a neutral separation between the researcher and the research object. Please, check the first author’s reflexivity on Supplementary material 6.

The fundamental unit of raw data analyzed to derive meanings, identify common patterns, and construct themes in this study was a sentence. Due to the sensitive nature of the research, participants did not consent to the sharing of their complete data (whole transcripts) in a public repository. However, Supplementary materials are accessible online, as previously indicated.

## 3 Results

The following results are derived from data collected in a context that is not inherently “natural” or observational. Rather, these findings are drawn from explanatory and argumentative narratives, which mirror the personal reasoning methods, assumptions, and references of legal actors when confronted with themes and issues typical in decision-making scenarios. Nevertheless, these results shed light on variables that can significantly influence the decisions of legal professionals in child custody cases. Additionally, the cognitive strategies outlined below remained consistent across both the Brazilian and English samples, as well as among different professionals. Any meaningful differences and pertinent discussions are elaborated in item 4.1.

For the sake of the readership, the full content of themes’ excerpts is available in Supplementary material 7. Participants’ quotations are associated with their ID, comprising their country (‘BR’ for Brazil; ‘EN’ for England) and category (‘Jd’ for Judge; ‘Lw’ for Lawyer; ‘Pr’ for Prosecutor; ‘Psy’ for Psychologist; ‘SW’ for Social Worker). Additionally, Brazilian participants have their city indicated in their ID (‘BsB’ for Brasília; ‘POA’ for Porto Alegre; ‘SP’ for São Paulo).

Throughout the data analysis process, we noticed that the themes generated could be divided into two domains, considering their characteristics and their content within the decision-making process towards uncertainty traits. We identified the first domain as ‘heuristics’ and themes belonging to this domain indicate strategic knowledge that is applied to select “operators that are most likely to lead to the goal state” (van Gog et al., 2005, p. 237). They are a cognitive mechanism used to search the problem space and make decisions within the task environment (Hutchinson and Gigerenzer, 2005). They provide ‘rules of thumb’: guidelines based on practice rather than theory (Shah and Oppenheimer, 2008). The heuristic themes identified in this study illustrate how legal professionals navigate uncertainty by applying strategic knowledge. This strategic knowledge guides them in deciding which information to select within the decision-making context, how to evaluate this information, the limitations associated with the selection and evaluation of information, and potential referrals to external court sources that can assist in resolving or solving the case. We identified four types of heuristics in this study: (a) selection; (b) evaluation; (c) degrees of freedom; and (d) outsourcing decisions and resolution.

The second domain identified was ‘metacognition’, and themes within this domain resemble metacognitive knowledge applied as a supervising and monitoring aid. This metacognitive knowledge functions in the “process of selection and application of operators by keeping track of the progress toward the goal state” (van Gog et al., 2005, p. 237). This type of strategy ensures that decision-makers feel comfortable with their decisions and actions throughout the decision-making process. This is crucial because decisions made in natural settings often involve high stakes (Orasanu and Connolly, 1993; Patterson et al., 2016). The metacognitive themes identified in this study elucidate how legal professionals navigate uncertainty by applying metacognitive knowledge. This knowledge aids them in monitoring and supervising their actions and intermediary decisions throughout the process, ensuring that they can be confident that their actions align with what makes sense or is deemed ‘the right thing to do’ in a given case. This aligns with priorities and importance for them in the decision-making process. We identified three types of metacognitive strategies in this study: (a) custodial arrangements; (b) professional practices; and (c) BIC speech.

We understand that heuristics and metacognitive domains represent cognitive strategies employed by legal professionals to grasp uncertainty stemming from contextual issues. These strategies seem to be pivotal for enabling legal professionals to navigate and operate effectively within the decision-making environment. Hence, these cognitive strategies play a crucial role in mitigating the complexity of uncertain situations inherent in child custody cases. The subsequent presentation outlines both domains, shedding light on their distinctive characteristics and contributions to the decision-making process in the legal realm.

### 3.1 Heuristics in child custody decision-making

Our results indicate that when faced with the intricate uncertainties stemming from child custody context factors, legal professionals employ heuristics to streamline their selection, consideration, and analysis within this specific context. These heuristics function as guiding principles, directing attention toward potential solutions and facilitating the organization and comprehension of uncertainties within the case environment.

#### 3.1.1 Selection

We acknowledge that the initial step legal professionals take, when faced with uncertainty induced by contextual factors, is to meticulously examine the child custody context. This process involves selecting elements considered crucial and warranting primary consideration in the specific case at hand. Consequently, ‘selection heuristics’ serve to prioritize aspects deemed important, guiding the exploration and consideration of key factors during the decision-making process.

In this study, legal professionals outlined heuristics for selecting issues related to: (a) *the child’s basic needs* (“[I examine] all aspects related to the fundamental needs [of the child]…housing, physical well-being, clothing, food” BR_BsB.SW.02); (b) *the child’s mental health and perception of stability* (“post-separation, the emotional impact on a child is a genuine concern” EN_Lw.02; “I think that kind of stability is the foundation, the bedrock of the child’s life, and you do not disturb it. They need their interests and stability outside of the parental relationship” EN_SW.01); (c) *the child’s emotional bonds* (“[to protect] this coexistence with the other parent or even with other family members is important and is a child’s right” BR_Pr.03); (d) *the child’s family dynamic* (“interviews are conducted individually with each parent and sometimes with other relatives to help us understand how the family is organized and their dynamic as a family” BR_POA.SW.01); and (e) *the parents’ co-parenting skills* (“you look at the parenting, the parents’ relationship styles, and whether they are sensitive to the needs of the child or unresponsive, avoiding or neglecting the needs of the child” EN_SW.04). All these issues encompass the child’s developmental needs and strongly support the association between the child’s best interests and their developmental needs, as emphasized by one participant: “we try to discern the child’s needs at each step of their development” BR_BsB.SW.01.

#### 3.1.2 Evaluation

Following the identification of crucial priorities in a given case, legal professionals proceed to determine how they will evaluate the gathered information. This involves the utilization of ‘evaluation heuristics’, which establish general principles and guidelines based on practical experience rather than theoretical frameworks. These general evaluation principles may encompass:

a) *No need to hear the child*: “I do not see any active participation [of the child] in order to help us make a decision (BR_BsB.Jd.02); “what is terrible is that the child almost gets ignored as a person in the dispute between the parents” (EN_Lw.06); “ideally, the child should not know that there are court proceedings” (EN_Jd.03); “that is, when the adults agree, we do not ask what the best interests of the child are. It is assumed that if the adults are on the same page, BIC is preserved, from this perspective” (BR_SP.Psy.01);
b) *The older, the better*: “I confess that I prefer to talk with adolescents than with young children” (BR_POA.SW.02); “especially if it’s a child over age 8, 9, 10, when they are able to express their views. It’s very difficult when they are very small, at 1, 2, 3, 4 years-old, the decision is made purely then from an objective perspective, and maybe an expert might say as to what is in their best interest” (EN_Lw.03);
c) *Children as subjects of rights*: “I think there is the issue of the child being seen as a subject, as someone who feels, that can participate, who has an opinion and understands what is happening. I think it [the child’s role] has to be an active role, [they are] a protagonist for me, I think the child has to speak” (BR_BsB.Psy.02);
d) *Trading-off interests*: “In child custody cases, you have to weigh what is the best interest of that child concerning the type of custody, the coexistence arrangement, alimony, etc...” (BR_Pr.01).

In the evaluation process, certain legal professionals, such as judges, lawyers and prosecutors, do not consider the child as an active agent, often citing parental agreement as grounds for excluding the child from the proceedings – see Features CS2.1, CS2.2 and CS4.5 in Supplementary material 7. Additionally, older children seem to ease proceedings as their communication skills obviate the need for specialized training. In England, besides the age criterion, some legal professionals assess the child’s ‘Gillick competence’ to determine their psychological maturity and ability to comprehend all the circumstances and implications.^3^

When involving a child in the evaluation process, legal professionals in both countries typically comprehend the child’s meaningful relationships, encompassing family, school, and friends. They also explore the child’s perspective on their family and the conflict itself to gain insight into their routine and the individuals involved in it. To capture this information, psychosocial staff commonly engage in activities such as playing games, constructing a genogram (a graphic representation of the family generations and the relationships between family member, specifically used by Brazilian psychosocial professionals – see McGoldrick et al., 2008), or employing drawing exercises to interact and communicate with young children while observing their behavior. Another strategy, mentioned solely by Brazilian experts, involves visiting the family household to observe the child in their natural context.

During the decision-making process, certain needs, such as the child’s emotional bonds and the right to family coexistence, are deemed more critical than others. However, these priorities may be overridden if there is a threat to the child’s physical or mental integrity. Recognizing the dynamic interplay among various aspects of the child’s needs, some legal professionals believe that weighing and trading off these needs is a crucial step in the decision-making process.

#### 3.1.3 Degrees of freedom

After identifying crucial aspects within the child custody context and applying general principles to evaluate the information, legal professionals tend to recognize the limits of their actions and decisions by employing ‘degrees of freedom’ heuristics. The main limitations identified by participants included: (a) *the welfare checklist*: in England, it is the primary constraint for determining the child’s best interests, as stated in Section 1 (3) of the Children Act 1989. A legal professional in England emphasized its significance, stating that, “perhaps in Britain, the best interests of the child are represented by the welfare checklist... So, for me, I always refer to the welfare checklist” (EN_SW.03), and another mentioned, “I tend, certainly, on a difficult case, to go through each element of the welfare checklist quite slavishly” (EN_Jd.01); (b) *prescriptions from law*: for some Brazilian legal professionals, joint custody should be awarded because that is what the law dictates, especially in the absence of an agreement between parents. A Brazilian legal professional stated that “today, the law determines that legal custody is joint custody. Moreover, the law states that the rule is [to award] joint custody” (BR_BsB.Jd.02), and another emphasized, “the law is clear, without agreement between the parents, the custody will be joint, except in rare hypotheses” (BR_POA.Jd.01).

In both countries, the legal framework plays a significant role in shaping how legal professionals perceive and evaluate the decision-making environment. These ‘legal constraints’ are crucial for providing structure and reliability to the decision-making process. However, there is a concern in Brazil where legislation outlines joint custody as the default arrangement, regardless of the unique aspects of each case and the specific needs of the child. This legal constraint may lead legal professionals to overlook the uncertainty and complexity inherent in determining the child’s best interests. In contrast, the English welfare checklist emphasizes the child’s individuality and the importance of adopting a holistic perspective in child custody matters.

#### 3.1.4 Outsourcing decisions and resolution

After acknowledging limitations within their practices, some legal professionals strive to resolve cases outside the court, particularly when it can lead to less acrimony or a swifter resolution. They achieve this by employing heuristics that guide them to outsource decisions and resolutions. In this study, legal professionals’ accounts point to specific issues:

a) *Extra-judicial information*: seeking information from institutions and social protection services. Legal professionals in both countries highlighted the school as the primary source of information outside the legal environment: “the school is a great indicator; school performance and the child’s behavior at school are indicators of the child’s needs, the problems they are facing, and how these problems are presenting themselves” (BR_Pr.01); “[I] always talk to professionals, if possible, who knew the child... usually, that would be a school teacher or the head of the school, who would give us some sort of insight into how the parents’ dispute… because there’s always a dispute involved, how it [parental dispute] is affecting the child” (EN_SW.01);
b) *Self-arrangements*: another way to outsource decisions, avoiding parental litigation, is by empowering the family. Encouraging parents to actively participate in decision-making is seen as a way to preserve the child’s interests and contribute to the overall well-being of the family: “we have to try to give back their ability to solve their issues. ‘*Oh, it is in your hands*,’ we say, ‘*we will inform the judge, but the power [to find a better solution] is in your hands*’. Everything goes through the parents” (BR_BsB.SW.02); “the court is not going to be there forever; the court is not going to be involved in their lives in every decision, so if upon separation we can get them to work together and come up with a plan together, then that kind of works for the future of the child” (EN_Lw.01). The underlying rationale is that the State should refrain from disrupting a functioning family dynamic, as imposed modifications could potentially worsen the situation. Additionally, fostering the independence of the family from judicial intervention was considered beneficial.
c) *Mediation and conciliation:* legal professionals emphasize the importance of alternative dispute resolution methods, such as mediation, conciliation, or treatment, as effective means to avoid litigation that could potentially harm the child’s best interests. One professional from Brazil highlighted these options, stating, “mediation, conciliation… these things that can solve the situation without having to judicialize the issue” (BR_BsB.Psy.02). Similarly, an English legal professional underscored the role of mediation, noting, “so, mediation is an opportunity for parents to try to solve their problems together. Without somebody, a stranger or a judge, for instance, having to make decisions on their behalf” (EN_Lw.04). This approach aligns with the goal of promoting collaborative resolution methods that empower parents to actively participate in decision-making processes and prioritize the child’s well-being.

### 3.2 Metacognitive strategies in child custody decision-making

Our data reveal that legal professionals employ not only heuristics, guiding them in the selection, evaluation, and outsourcing of information but also metacognitive strategies. These metacognitive approaches are designed to instill confidence in legal professionals, providing certainty about their decisions and actions throughout the decision-making process. Although the primary objective is generally oriented toward the child’s best interests, these strategies may also serve the professionals’ self-protection. In essence, metacognitive strategies play a crucial role in cultivating a sense of assurance among legal professionals as they navigate the intricate landscape of child custody cases.

#### 3.2.1 Custodial arrangements

This metacognitive strategy significantly shapes the perspectives and preferences of legal professionals regarding a specific type of custodial arrangement. Some Brazilian legal professionals lean towards viewing joint custody as an optimal arrangement. They perceive it not only as aligned with the child’s best interests but also as a mechanism to establish a power balance between parents: “I understand that joint custody is the one that best meets [the child’s best interests], precisely because it offers a balance of power in the exercise of family authority” (BR_BsB.Jd.01). This metacognitive strategy may reinforce decisions due to legal constraints related to joint custody in Brazil.

For some professionals, joint custody is seen as a means to spare the child from becoming a bargaining object between parents. Regardless of issues in the co-parenting dynamic, some professionals advocate for joint custody as a way to put both parents on equal footing: “[the legislator wanted to say:] It does not matter if you do not get along... the custody is joint, make it work, you will have to figure it out. It puts both parents on an equal footing. I think that is the most important thing” (BR_POA.Psy.02). However, there are varying perspectives, with some professionals, including those in England, suggesting that joint custody should be conditional: “I think if you have eliminated any particular risks for the child, then I think joint custody is always going to be the best outcome, for the best interests of the child. Because children thrive the more people they have involved” (EN_Psy.08). Overall, these professionals believe that establishing joint custody should be contingent on factors such as mutual respect between parents, effective co-parental communication, the ability to share decisions, and the absence of significant risks to the child.

#### 3.2.2 Professional practices

This metacognitive strategy involves the supervision and monitoring of legal professionals’ practices. Some Brazilian psychologists assert that the concept BIC is not extensively discussed within the field of psychology, as it is considered a legal term: “I think that within psychology no one discusses this [BIC]” (BR_BsB.Psy.03); “Actually, we do not use that term [BIC] much. It’s a legal term” (BR_BsB.Psy.01).

When lawyers become active participants in parental disputes, some professionals argue that this can lead to increased acrimony between parents, potentially prolonging the dispute. As one Brazilian professional pointed out, “there are times that lawyers do not help because they have those interests, interests in continuing the litigation, they want the fight because then they will have some financial benefit from it” (BR_SP.Jd.01). Another professional in England expressed: “it’s very easy as lawyers to become aggressive, to become overly involved in a case to the point where all you are doing is being a mouthpiece for your client” (EN_Lw.04). Some lawyers admit to prioritizing their clients (parents) over the child’s best interests, focusing on subjective opinions rather than what is truly in the child’s best interests. For instance, one English lawyer stated, “my role is not to promote what is in the best interest of the child, my role is to advise my clients as to whatever their subjective opinion is, how would it be received by the law?” (EN_Lw.02).

However, there are legal professionals who emphasize the importance of putting BIC first and educating parents on child-centered decision-making: “the role of the lawyer in the context of the best interests of the child is to make the parents understand that the custody dispute does not concern them, it concerns the child” (BR_BsB.Lw.01); “I got to keep bringing them back down to the basics: this is about the child and it is a child-centered, child-focused decision that the courts make; it’s not about what you think, it’s not about what the other party thinks, this is about what is in this child’s best interests” (EN_Lw.03). As seen, the emphasis is on fostering an understanding that the custody dispute is fundamentally about ensuring what is best for the child involved. This child-centric approach, as advocated by these professionals, aims to create a shift in perspective and prioritize the welfare of the child over other considerations.

Urged by the metacognitive strategy that takes place to ensure that legal professionals are doing their best or what is right for the child under dispute, they engage themselves in ‘active practices’. These practices involve educating and guiding parents on maintaining a less acrimonious dialogue and increasing awareness about the impact of disputes on the child. For instance, professionals reported: “we try to guide the parent on how to have a less aggressive dialogue. Some techniques that we try to teach to these parts [parents]” (BR_Pr.02); “[we try to] educate them [parents] really about the effects on the child of an acrimonious dispute” (EN_Psy.07). Through these practices, legal professionals aim to ensure the child’s welfare and interests are prioritized and protected, irrespective of the particular custodial arrangement that will take place.

Additionally, some legal professionals use evaluation services to monitor and supervise their decisions and actions. In Brazil, there is a debate about whether professionals should deliver interventions during evaluations or maintain an observational role: “[the psychosocial professionals’ role] is to promote reflection, and intervention in some cases, where we perceive cases of vulnerability or risks that are spotted and referred to the support network” (BR_BsB.SW.02); “when they [parents] come for an evaluation, they come very much in a position of defense. Therefore, I think it is a bit of an illusion for us to think that there will be an intervention, a big intervention. We can suggest interventions, of course, but our role here is evaluating” (BR_POA.Psy.02). In England, some view the work of the ‘Children and Family Court Advisory and Support Service – CAFCASS’ (the national agency responsible for evaluations in child custody cases in England) as risk-averse, potentially prioritizing the safest route even if it may not be in the best interests of the child: “I do think that they [CAFCASS] are a very risk-averse organization. They certainly have become that. So, for instance, they will always take the safest route, safest route even if it means that a child potentially might suffer by not having a relationship” (EN_Lw.04).

#### 3.2.3 BIC speech

This metacognitive strategy involves leveraging BIC to qualify or justify decisions and actions within the decision-making process. Some legal professionals may seemingly prioritize the parents’ interests while asserting that they are safeguarding the child’s welfare, as evidenced by the statement: “you do not say that. But, some parents are motivated by money. Money is really important to determine what is in the best interests of your child. Trust me, money!” (EN_Lw.02). Alternatively, BIC might be used merely rhetorically: “it seems to me that this expression [the ‘best interests’] is utilized more as a figure of speech than to express thorough preoccupation. It sounds good when you say ‘best interests of the child’. (…) It seems that the problem would be solved just by mentioning it [BIC]” (BR_SP.Psy.03).

Nevertheless, some lawyers endeavor to strike a balance between prioritizing the interests of both the parents and the child: “I try to achieve the outcome that my client wants, which should be linked back to what is the child’s best interests. […] But, ultimately, it is not for me to determine what’s in the child’s best interests, it’s for the client to determine with my advice and then we could forward their position” (EN_Lw.01). This illustrates an attempt to reconcile both perspectives by addressing the child’s interests through the viewpoint of the client (parent).

As seen, the ‘BIC Speech’ is a metacognitive strategy that serves as a self-assurance mechanism for legal professionals. It points out a potential discrepancy where some professionals may seem to prioritize the parents’ interests while asserting that they are acting in the child’s best interests. Additionally, we notice the rhetorical use of BIC, where the term may be mentioned without a thorough examination of its implications.

## 4 Discussion

NDM aims to comprehend how real-life decisions unfold in challenging, real-world scenarios characterized by uncertainty, high stakes, and constraints within teams and organizations (Klein, 2008; Hoffman and Klein, 2017). This approach focuses specifically on “how expert practitioners perform cognitively complex functions” (Patterson et al., 2016, p. 229). We believe the results of this study align with these principles. Experts, in this case legal professionals dealing with child custody cases, encounter and navigate uncertainty, high stakes, and limitations within team and organizational contexts. Additionally, these legal professionals use cognitive strategies to effectively manage the challenges posed by uncertainty in their decision-making processes.

Legal professionals seem to start by employing heuristics to select decision topics (e.g., “what should be addressed to improve the child’s best interests?,” “what should be assessed?”) and evaluate relevant information (e.g., “how should I analyze this?,” “what is the suitable way to analyze this?”). They also acknowledge the limitations of their actions and decisions within specific cases (e.g., the family will not be able to fit into this arrangement). Depending on how information is selected, evaluated, and constrained by legal, team, and organizational issues, legal professionals can access sources outside the court to ‘cool down’ or even resolve the cases. In parallel, legal professionals employ metacognitive strategies to feel secure and confident about their decisions/actions, aiming to preserve either the child’s best interests or their own. This process illustrates the intricacies of the decision-making process in child custody cases, providing insight into how it is structured amid contextual dynamics and constraints.

The results indicate that the inherent uncertainty in child custody cases prompts legal professionals to deploy strategic approaches. Heuristics emerge as a crucial aid, allowing legal professionals to selectively choose and evaluate important information guided by specific principles (Rehak et al., 2010). These cognitive shortcuts streamline the decision-making process by focusing on key aspects essential for evaluating the child’s best interests, alleviating cognitive load and facilitating quicker, more pragmatic decisions. However, a disadvantage of employing heuristics is the potential loss of accuracy, given that they often go unnoticed by decision-makers (McCray et al., 2002).

In addition to heuristics, metacognitive strategies ensure the validity and adherence to specific actions, practices, and principles throughout the decision-making process. This self-assurance mechanism reflects a nuanced interplay of cognitive strategies employed by legal professionals to navigate the multifaceted and uncertain realm of child custody cases (Mendes and Bucher-Maluschke, 2017a; Mendes and Ormerod, 2023).

As legal professionals use these higher-order cognitive strategies for self-monitoring and assurance throughout the decision-making process, they may ensure that their practices and principles align with the child’s best interests. Conversely, they may employ these strategies to act against the child’s best interests while providing themselves with the perception that they are acting benevolently – as seen in the case of the ‘BIC Speech’ strategy.

The BIC issue illustrates the interplay between contextual uncertainty and cognitive strategies, which is exemplified by the practice of some legal professionals to evade hearing the child (in court or via evaluations) when a parental agreement exists. This implies that parental consensus equates to preserving the child’s best interests, suggesting two key points: (1) the link between this evaluation heuristic and other elements of child custody cases, such as difficulties or limitations when attempting to hear the child (i.e., degrees of freedom), and the need to cease parental litigation by means of reaching an agreement (i.e., outsourcing decisions and resolution); and (2) ‘what is the best for the child’ in child custody cases is an adult-centric interpretation of what adults perceive as essential or prioritized for the child. Consequently, the child’s interests are often filtered through adult perspectives (parents, legal professionals) and influenced by practical considerations, such as evaluator capabilities, scope of inquiry, judicial limitations, and lawyers’ professional practices (Mendes and Ormerod, 2019; Mendes, 2024).

All these issues illustrate the role played by heuristics and metacognitive strategies in delineating the contours of the decision-making process within the context of child custody. We understand that both heuristics and metacognitive strategies emerge as instrumental tools, facilitating legal professionals in navigating the pervasive uncertainty inherent in such cases. However, it is crucial to underscore that, while heuristics and metacognitive strategies offer valuable cognitive aids, their efficacious deployment is contingent upon the possession of proper training and awareness among legal professionals. The results highlight the potential for these cognitive strategies to be misused in the absence of such training. Specifically, there is a risk that these strategies might be used to suppress, rather than address, the inherent uncertainty of child custody cases.

Suppressing uncertainty, rather than acknowledging and addressing it properly, is recognized as a non-effective decision-making approach (Lipshitz and Strauss, 1997; Lipshitz et al., 2001). In child custody cases, adopting such an approach may increase uncertainty and put the well-being of children and families at risk (Mendes, 2022; Mendes and Ormerod, 2023). Instead, we advocate for recognizing the sources of uncertainty to comprehend their potential impact on the decision-making process. This understanding allows for evidence-based responses to these sources, thereby mitigating uncertainty and leading to child-focused decisions and outcomes. Therefore, the necessity for continuous training and heightened awareness is underscored, ensuring that legal professionals employ heuristics and metacognitive strategies judiciously, ethically, and with a nuanced understanding of their implications in the realm of child custody decision-making.

### 4.1 Main differences between Brazil and England

In Brazil, an example of limitations that shape the decision-making process is the use of a sole or joint custody decision, which is a default by law (Mendes and Ormerod, 2021). In England, the decision-making outcome is not dichotomous as in Brazil. English family courts award ‘child arrangements’ that concern who the child should live with and the time they should spend with the non-guardian or non-residential parent, without a mandatory custodial arrangement like in Brazil (Mendes and Ormerod, 2021). Another contrasting characteristic is how English professionals adhere to the welfare checklist presented in Section 1(3) of the Children Act 1989. They “always refer to it” (EN_SW.03) and tend to use it in the most difficult cases, where they use it “quite slavishly” (EN_Jd.01). In Brazil, BIC is enacted by its Constitution’s Article 227° (and by the Children’s and Adolescent’s Statute), but Brazilian professionals constantly refer to BIC as vague or as not part of their practice (Mendes and Ormerod, 2019; Mendes, 2022, 2024).

The observed discrepancies are significant, especially considering the stringent framework provided by the Brazilian Civil Law, which includes comprehensive norms, regulations, and instructions (Mendes and Ormerod, 2021), offering legal professionals a structured and readily accessible environment for the child custody decision-making process. Paradoxically, Brazilian legal practitioners resort to “workarounds,” such as educating parents, highlighting an inadequacy in the law’s guidance for efficient decision-making and safeguarding the child’s interests (Mendes and Ormerod, 2021, 2023). In contrast, English legal professionals operate within a Common Law system grounded in case law and precedents, which ostensibly suggests a more flexible and unstructured milieu. However, they heavily rely on statutory provisions, notably the Children Act 1989 (CA 1989), and meticulously align the child’s best interests with the welfare checklist, which is presented in the Section 1 (3) of CA 1989. This indicates a strategic adaptation by legal professionals to mitigate uncertainties within their respective legal systems. In the case of Brazil, a strict and formalized system is complemented by strategies that introduce discretion (“workarounds”) into legal decision-making. In the case of England, a less formal case law system is complemented by strategies and systems (e.g., the welfare checklist) that introduce additional rigor and consistency.

### 4.2 Highlights and potential contributions

Unveiling the intricate cognitive processes at play within child custody decisions is crucial for understanding how legal professionals navigate this complex domain. This study, informed by the NDM framework, delves into these demanding processes, positing that child custody decision-making can be conceptualized as a dynamic tug-of-war between contextual uncertainties and legal professionals’ cognitive strategies.

On one side, contextual uncertainties exert a powerful pull. Family dynamics, crises, developmental issues, organizational constraints, and judicial limitations create a highly pressurized decision-making environment (Mendes and Bucher-Maluschke, 2017a; Mendes and Ormerod, 2023). Legal professionals must grapple with these multifaceted uncertainties, seeking to understand the nuances of each case and the potential ramifications of their decisions. On the other side, legal professionals counterbalance these uncertainties with their cognitive strategies. The constant interplay between these opposing forces shapes the trajectory of each case. The unique singularities of each family and situation ultimately determine the final resolution, highlighting the dynamic and individualized nature of child custody decision-making process.

By examining how legal professionals navigate the highly complex and demanding environment of child custody cases, we gain valuable insights into their thought processes and decision-making mechanisms. Furthermore, the identified steps – Selection, Evaluation, Degrees of Freedom, and Outsourcing – can potentially constitute a comprehensive NDM process model, mirroring the real-world decision-making steps involved in reaching child custody decisions. This is relevant as, traditionally, NDM has been concentrated on decision-making processes in military, firefighting, aviation, and human-machine interfaces (Klein, 2008; Gore et al., 2015; Patterson et al., 2016). The application of NDM principles to child custody cases broadens its scope into a critical and complex real-world domain, reaching the far ends of the socio-technical systems spectrum. It specifically extends toward the “socio” and intentional systems as opposed to systems governed by physical principles.

To our knowledge, this is the first attempt to apply an NDM approach to child custody cases. We believe our contribution is twofold: (1) *broadening the scope of NDM:* by intricately delineating the child custody decision-making process, we showcase the versatility and significance of the NDM framework in comprehending intricate real-world domains beyond its conventional applications, such as physical systems; and (2) *prioritizing Child Welfare:* we showcase how legal professionals navigate the complexities of these cases and elucidate the methods and thoroughness with which they address the best interests of the child.

Additionally, we contend that this study establishes a foundation for the creation of NDM-based tools tailored for child custody decision-making. Such tools hold the promise of refining the decision-making process, fostering a more comprehensive and nuanced approach that places paramount importance on the best interests of children entangled in custody disputes.

### 4.3 Limitations and future directions

We acknowledge the inherent limitations in this study’s exploration of child custody decision-making through the NDM lens. The focus on legal and cultural elements within Brazil and England imposes constraints on the results’ transferability (Maxwell and Chmiel, 2014). To enhance the study’s applicability, future research could broaden its scope to encompass diverse cultures and additional countries governed by both civil and common law systems, potentially yielding richer insights. Moreover, while the results provide insight into how decision-makers navigate uncertainty in child custody cases, a deeper investigation is needed to fully understand the intricate interplay between cognitive strategies and real-time decision-making. Exploring how decision-makers “map” these strategies would offer invaluable knowledge for refining the NDM model and potentially developing targeted interventions in this critical domain.

It is further suggested that the impact of variables significantly influencing the cognitive reasoning of legal professionals, and their subsequent effect on the decision-making process, be studied in greater depth. These variables include the age and maturity of the children, the characteristics of the conflict (such as its intensity and the degree of communication and cooperation between parents), and negative criteria of parenthood (such as psychopathology).

By acknowledging these limitations and proposing avenues for future research, this study highlights the potential of the NDM framework to showcase complex decision-making processes in varied contexts. It sets the stage for further exploration that can contribute to more informed and child-centric outcomes in child custody cases worldwide.

## Data availability statement

The original contributions presented in the study are included in the article/Supplementary material, further inquiries can be directed to the corresponding author.

## Ethics statement

The studies involving humans were approved by University of Sussex’s Social Sciences & Arts Research Ethics Committee under the Certificate of Approval number ER/JA454/2. The studies were conducted in accordance with the local legislation and institutional requirements. The participants provided their written informed consent to participate in this study. Written informed consent was obtained from the individual(s) for the publication of any potentially identifiable images or data included in this article.

## Author contributions

JM: Conceptualization, Data curation, Formal analysis, Funding acquisition, Investigation, Methodology, Project administration, Resources, Software, Validation, Visualization, Writing – original draft, Writing – review & editing. TO: Conceptualization, Methodology, Supervision, Writing – original draft, Writing – review & editing.

## Footnotes

^1^Throughout this paper, despite legal and definitional differences, ‘divorce’ and ‘parental separation’ will be referred to as the same thing: the relationship breakdown between two people who had a child together.

^2^Although ‘custody’ remains a legal term within Brazilian law, within English Law concerning private law cases, ‘custody’ is not referred to anymore. Since the Families Act 2014, the correct term is ‘child arrangement’ and it addresses issues regarding the child after the breakdown of the marital relationship. Nonetheless, we use ‘custody’ as uniform term to designate in both Brazil and England: (a) where the children will live; (b) how much time they will spend with each parent; and (c) access to the child: arrangements for parents to visit/see their children after parental separation. For further discussion, see Mendes and Ormerod (2021).

^3^‘Gillick competence’ emerged in the context of medical treatment involving children under 16 years old and consent. A child is Gillick competent if they present enough maturity and intelligence to understand the nature and implications of the situation. This principle has been extended to any legal matters in which the child’s wishes and feelings might be important for the decision-making process. For further discussion, see Griffith (2016).
