# Supplementary material for: Making sense out of uncertainty: cognitive strategies in the child custody decision-making process
Source: Front Psychol. 2024 Jul 15;15:1387549. doi: 10.3389/fpsyg.2024.1387549 (PMC11284646; doi:10.3389/fpsyg.2024.1387549)
Supplement: Supplementary file 1 [file Table_1.pdf]

Supplementary Material 1

Article: Making sense out of uncertainty: cognitive strategies in child custody decision-making process

Journal: Frontiers in Psychology

Authors: Josimar Antônio de Alcântara Mendes; Thomas Ormerod

Interview Questions

Participant: Category: Country/City:

For how long have you been working in this field?

Understanding the concept of 'the best interests of the child' (BIC)

First, I would like to have an idea of how you understand the BIC in general, so I would like to know:

1) What do you understand by 'the best interests of the child' (BIC)?
2) There are some critiques arguing that BIC is too open, too subjective, too vague… what are your thoughts on it?

National and International Legislation on BIC

Now, I would like to know if you perceive the BIC related to national and international legislation, so:

3) How do you believe BIC is addressed by the legislation?
4) How do you think BIC relates to the child's rights?

BIC and Parental Separation Context and its Evaluation

Now, I want to discuss how BIC is related and applied to parental separation and custody context, so:

5) What are the child's interests that are at more risk in child custody disputes after parental separation?
6) How to evaluate BIC in child custody disputes after parental separation?
7) How to promote BIC in child custody disputes after parental separation?
8) What are the child custody disputes, after parental separation, in which the decision-making based on BIC is most difficult?

<u>Child's role in the BIC evaluation</u>

Now, I want to understand how the child is integrated into the child custody decision-making process, so:

9) What is the role of the child in the process of evaluation and promotion of their best interests in post parental separation disputes?
10) How the children's ideas, desires, and yearnings are taken into account during the decision-making process?
11) Is there any difference between kids' and adolescents' interests in in child custody disputes after parental separation?

<u>Parents' role in the BIC evaluation</u>

Now, I want to discuss how parents' relationship and their parenthood are taken into account during the child custody decision-making process, so:

12) How do you believe parents can affect a child's best interests in child custody disputes after parental separation?
13) In a situation of post parental separation dispute, what are the main aspects of the parent-child relationship that have to be taken into account to make a decision?
14) There is any kind of 'custodial arrangement' that fits better the best interests of the child after parental separation?

<u>Other BIC factors</u>

Now, I want to discuss other factors or characteristics that can impact the BIC and the child custody decision-making process, so:

15) Do you believe that the child's development is related, somehow, to the BIC? (probe)
16) What is the relationship between BIC and the child's social network (extended family, school, friends, community, etc.)?
17) **For judges:** how do you perceive the performance of the prosecutor, psychologist, social worker and lawyers in child custody decision-making? How can they act to ensure the best interests of the child? **For prosecutors**: how do you perceive the performance of the judge, psychologist, social worker and lawyers in child custody decision-making? How can they act to ensure the best interests of the child? **For psychologists:** how do you perceive the performance of the prosecutor, judge, social worker and lawyers child custody decision-making? How can they act to ensure the best interests of the child? **For social workers**: how do you perceive the performance of the prosecutor, judge, psychologists and lawyers child custody decision-making? How can they act to ensure the best interests of the child? **For lawyers:** how do you perceive the performance of the prosecutor, psychologist and social workers in child custody decision-making? How can they act to ensure the best interests of the child?
