# Supplementary material for: Making sense out of uncertainty: cognitive strategies in the child custody decision-making process
Source: Front Psychol. 2024 Jul 15;15:1387549. doi: 10.3389/fpsyg.2024.1387549 (PMC11284646; doi:10.3389/fpsyg.2024.1387549)
Supplement: Supplementary file 2 [file Table_2.pdf]

frontiers

Supplementary Material 2

Article: Making sense out of uncertainty: cognitive strategies in child custody decision-making process

Journal: Frontiers in Psychology

Authors: Josimar Antônio de Alcântara Mendes; Thomas Ormerod

Participants' Demographics

English participants' city is omitted to avoid any possibility of identification because, in many cases, there was just one participant per city. For the same reason, in Brazil, the prosecutors' city is also not displayed as participants were mainly from the same city and just one prosecutor was from another city.

Participants' Basic Sociodemographic Information Per Country

Country/City	Category	Gender	Years of Experience	Excerpt Reference Code	ID
				BR_BsB.Jd	
		F	12	01	P1
	Judges	F	5	02	P2
		F	5	03	P3
		M	3	04	P4
				BR_BsB.Lw	
		F	9	01	P5
	Lawyers	F	6	02	P6
Brazil/ Brasília		M	25	03	P7
				BR_BsB.Psy	
		F	13	01	P8
		F	18	02	P9
	Psychologists	F	18	03	P10
		F	15	04	P11
		F	10	05	P12
	Social Workers			*BR_BsB.SW*	
		F	13	01	P13

		F	18	02	P14
Brazil/ Porto Alegre				*BR_POA.Jd*	
	Judges	M	30	01	P15
		M	13	02	P16
				BR_POA.Lw	
	Lawyers	F	15	01	P17
		F	15	02	P18
		F	25	03	P19
				BR_POA.Psy	
	Psychologists	F	25	01	P20
		F	10	02	P21
		F	18	03	P22
		F	20	04	P23
				BR_POA.SW	
	Social Workers	F	3	01	P24
		F	3	02	P25
		M	2	03	P26
Brazil/ São Paulo				*BR_SP.Jd*	
	Judges	F	8	01	P27
		F	30	02	P28
		M	20	03	P29
		M	7	04	P30
				BR_SP.Lw	
	Lawyers	F	3	01	P31
		F	8	02	P32
		M	5	03	P33
		M	8	04	P34
				BR_SP.Psy	
	Psychologists	F	26	01	P35
		F	11	02	P36
		M	31	03	P37
		M	3	04	P38
				BR_SP.SW	
	Social Workers	F	30	01	P39
		F	44	02	P40
		F	5	03	P41
Brazil				*BR_Pr*	
	Prosecutors	F	14	01	P42
		F	15	02	P43
		M	5	03	P44
		M	6	04	P45
		M	2	05	P46
		M	16	06	P47
		M	25	07	P48
England				*EN_Jd*	
	Judges	F	30	01	P49
		F	12	02	P50

	M	16	03	P51
	M	23	04	P52
			EN_Lw	
Lawyers	F	5	01	P53
	F	20	02	P54
	F	04	03	P55
	F	28	04	P56
	F	03	05	P57
	F	10	06	P58
	M	08	07	P59
			EN_Psy	
Psychologists	F	26	01	P60
	F	14	02	P61
	F	11	03	P62
	F	9	04	P63
	M	30	05	P64
	M	9	06	P65
	M	14	07	P66
	M	12	08	P67
	M	24	09	P68
			EN_SW	
Social Workers	F	9	01	P69
	F	28	02	P70
	F	26	03	P71
	M	14	04	P72
	M	28	05	P73
**TOTAL**	F= 47 (64.4%); M= 26 (35.6%)	μ= 14.9 (SD=9.4)	-	73
