# Supplementary material for: Making sense out of uncertainty: cognitive strategies in the child custody decision-making process
Source: Front Psychol. 2024 Jul 15;15:1387549. doi: 10.3389/fpsyg.2024.1387549 (PMC11284646; doi:10.3389/fpsyg.2024.1387549)
Supplement: Supplementary file 3 [file Table_3.pdf]

Supplementary Material 3

Article: Making sense out of uncertainty: cognitive strategies in child custody decision-making process

Journal: Frontiers in Psychology

Authors: Josimar Antônio de Alcântara Mendes; Thomas Ormerod

Phase II (Open Coding): List of Codes

Code	Description	Units Of Analysis (Interviews)	Units Of Coding (Occurrences)
Assessing BIC[¥]	Strategies, methods and/or tools to assess BIC	55	188
Psychosocial Evaluation	Procedures, strategies, methods or tools used by psychologists and/or social workers to assess families (parents/children)	36	165
Hearing the Child	Procedures, strategies, methods or tools to hear children	51	160
Joint Custody	Issues regarding joint custody	50	148
BIC Hindering	Factors, characteristics, dynamics and/or events that might hinder BIC	52	115
BIC Definition	Any definition or description for BIC	52	98
Lawyers' Practice	Procedures, strategies, methods or tools carried by lawyers that characterise their practice	16	83
Parental Conflict	Statements regarding parental conflict	44	83
Contextual Needs	Statements regarding the child's context developmental needs	39	82
Material-physiological Needs	Statements regarding the child's material-physiological developmental needs	37	73
Challenges	Statements regarding factors,	42	72

Code	Description	Units Of Analysis (Interviews)	Units Of Coding (Occurrences)
	characteristics, dynamics and/or events that can appear as a challenge for legal actors		
Adolescents	Statements regarding factors, characteristics, dynamics and/or events related to adolescents	34	70
Best Child Arrangement	Statements regarding the best child custody arrangement	46	68
Decision-making context	Statements regarding factors, characteristics, dynamics and/or events related to the decision-making context	26	58
Judiciary & Law	Statements regarding factors, characteristics, dynamics and/or events related to the judiciary and/or laws	31	58
Psychologists' Practice	Procedures, strategies, methods and/or tools carried by psychologists that characterise their practice	18	57
Expert Witness - Independent	Procedures, strategies, methods and/or tools that characterise independent expert witnes' practice	22	54
Parental Alienation	Statements regarding 'parental alienation'	33	54
Child Involvement (in the parental conflict)	Statements regarding factors, characteristics, dynamics and/or events related to the child involvement in the parental conflict	29	52
BIC & Development	Statements addressing the relationship between BIC and child development	41	51
Empowering Parents	Statements regarding factors, characteristics, dynamics and/or events related to parental empowerment	23	49
Stability	Statements regarding factors, characteristics, dynamics and/or events related to the continuity or stability elements linked to the child development	25	49
Parenthood Vs. Conjugality	Statements regarding factors, characteristics, dynamics and/or events related to the relationship between parental roles and conjugal roles	32	48
BIC Characteristics	Any characteristics attributed to BIC	30	47
Child's Role	Statements regarding factors,	27	45

Code	Description	Units Of Analysis (Interviews)	Units Of Coding (Occurrences)
	characteristics, dynamics and/or events related to the relationship between parental roles and conjugal roles		
Decision-making process	Statements regarding factors, characteristics, dynamics and/or events related to the decision-making process	18	45
Family Coexistence & Contact Child	Statements regarding factors, characteristics, dynamics and/or events related to the maintenance of the family bonds and the contact of the child with both parents	28	44
Applying BIC	Procedures, rules and/or characteristics for the application of BIC	26	38
BIC & Family and Community	Factors, characteristics and/or dynamics related to the relationship between BIC, the family and the community	22	37
BIC & Child's Rights	Factors, characteristics and/or dynamics related to the relationship between BIC and the child's rights	26	36
Gender	Factors, characteristics and/or dynamics related to gender issues	16	36
CAFCASS	Factors, characteristics, dynamics and/or events related to CAFCASS	14	35
Educating Parents	Procedures, rules and/or characteristics related to psychoeducation, orientation and/or guidance to parents	20	32
BIC & Legislation	Statements regarding the relationship between BIC and legislation	23	31
Lawyers' Practice (Others' Perception)	Procedures, strategies, methods and/or tools pointed by other legal actors, which characterise lawyers' practice	11	29
Judges' Practice	Procedures, strategies, methods and/or tools carried by judges that characterise their practice	10	27
The decision made	Statements regarding factors, characteristics, dynamics and/or events related to the decision made by the court	16	26
Social Worker's Practice	Procedures, strategies, methods and/or tools carried by social workers that	9	25

Code	Description	Units Of Analysis (Interviews)	Units Of Coding (Occurrences)
	characterise their practice		
50-50 Arrangement (time divided equally)	Any conceptualisation, definition, description or statement regarding the equal-division-of-time type of child custody arrangement after parental separation	18	24
Legal Actors' Biases	Statements regarding legal actors' biases	17	23
Family Crisis	Statements regarding factors, characteristics, dynamics or events related family developmental crisis	16	22
Judges' practice (others' views)	Procedures, strategies, methods or tools pointed by other legal actors, which characterise judges' practice	9	22
Mediation	Statements regarding family mediation practices	11	21
Child as a Subject	Statements regarding the assumption and/or necessity to see and/or reinforce the child as an active subject of rights	12	19
Limitations	Statements regarding factors, characteristics, dynamics or events related to decision-making limitations	8	16
Legal Actors' Practice	Procedures, strategies, methods or tools carried by legal actors that characterise their practice	12	14
Future-oriented BIC	Statements addressing BIC's understanding and/or application that considers the future	5	11
Types of Arrangement	Statements addressing types of child custody arrangement	7	10
Psychologists' Practice (other's views)	Procedures, strategies, methods or tools pointed by other legal actors, which characterise psychologists' practice	2	9
Social Worker's Practice (other's views)	Procedures, strategies, methods or tools identified by other legal actors, which characterise social workers' practice	3	8
Custody Dispute Scenario	Statements regarding factors, characteristics, dynamics and/or events related to the custody dispute scenario	4	7
Pre-hearing	Factors, characteristics and/or dynamics related to events before the first hearing in	4	6

Code	Description	Units Of Analysis (Interviews)	Units Of Coding (Occurrences)
	the court		
Trading-off Needs	Procedures, strategies, methods and/or tools used to trade-off child's needs	4	6
Parental Equal Rights	Factors, characteristics and/or dynamics related parental equal rights issues	4	5
Before going to the Judiciary	Factors or characteristics related to the family's dynamics and/or events before the family goes to the court	4	4
Child Maltreatment	Statements regarding factors, characteristics, dynamics and/or events related to child maltreatment allegations	1	3
After the Decision	Factors or characteristics related to the family dynamic and/or child welfare after the decision is made and the case is closed	2	2
Present-oriented BIC	Statements addressing BIC's understanding and/or application that considers the present	2	2
Protecting the Child	Procedures, strategies, methods or tools used preserve the child from the parental conflict	1	2
Prosecutors' Practice	Procedures, strategies, methods and/or tools carried by prosecutors that characterise their practice	2	2
Prosecutors' Practice (others' views)	Procedures, strategies, methods and/or tools pointed by other legal actors, which characterise prosecutors' practice	2	2
Religion	Statements regarding religious beliefs and/or issues related to the child custody dispute	1	2

[¥]The best interests of the child principle
