# Supplementary material for: Making sense out of uncertainty: cognitive strategies in the child custody decision-making process
Source: Front Psychol. 2024 Jul 15;15:1387549. doi: 10.3389/fpsyg.2024.1387549 (PMC11284646; doi:10.3389/fpsyg.2024.1387549)
Supplement: Supplementary file 4 [file Table_4.pdf]

Supplementary Material 4

Article: Making sense out of uncertainty: cognitive strategies in child custody decision-making process

Journal: Frontiers in Psychology

Authors: Josimar Antônio de Alcântara Mendes; Thomas Ormerod

Phase III (Generating Themes): List of Codes

This document presents the Phase III's outcomes. It is comprised of 15 candidate themes and 34 features that were based on the analysis of all initial (open) codes created in Phase II.

Candidate Theme 1: Strategies to Avoid 'BIC-Harming Parental Litigation'

General description: strategies that legal actors use to shield the child from the parental litigation dynamic.

Feature (a): Self-arrangement: empowering the family

Description: advocating for self-arrangements for child custody by prioritising the family's capacity and competency to know and understand its own reality and needs. Therefore, the best decision regarding child custody will be made by the family itself

Source: [P2, P4, P5, P7, P13, P14, P20, P24, P27, P31, P34, P35, P42, P43, P44, P46, P49, P50, P51, P52, P53, P54, P55, P72, P73]

Feature (b): Educating Parents

Description: the need to orientate and educate parents going through a child custody dispute. This process would address issues regarding positive and effective parenting skills, roles and duties

Source: [P1, P3, P5, P16, P37, P25, P26, P27, P39, P41, P42, P43, P46, P47, P49, P55, P57, P60, P66, P67, P70]

Feature (c): Mediation & Conciliation

Description: practices toward family mediation and/or conciliation

Source: [P9, P16, P20, P27, P28, P29, P31, P42, P43, P47, P50, P51, P53, P54, P56, P59, P63, P66, P58, P70, P72]

Candidate Theme 2: Promoting BIC in Custody Dispute Cases

General description: how legal actors preserve the child's biopsychosocial and emotional needs during the child custody dispute

Feature (a): Preserving basic (material-physiological) needs and rights

Description: provision of the child's basic needs related to material-physiological issues

Source: [P2, P3, P4, P5, P6, P7, P8, P9 P11, P12, P13, P14, P16, P17, P22, P24, P26, P27, P29, P31, P32, P33, P34, P39, P42, P43, P44, P45, P46, P47, P48, P49, P50, P53, P55, P59, P64, P65, P69, P70, P71, P73]

Feature (b): Enhancing the child's psychosocio-emotional well-being

Description: enhance the child's needs related to psychosocio-emotional issues

Source: [P2, P3, P4, P5, P6, P7, P8, P9, P11, P13, P14, P17, P20, P22, P26, P27, P29, P31, P33, P42, P43, P44, P45, P48, P49, P53, P54, P55, P56, P59, P63, P64, P65, P66, P70, P72, P73]

Feature (c): Preserving the relationship with both parents

Description: strategies to protect the emotional bond between parents and children

Source: [P1, P2, P3, P4, P7, P10, P11, P13, P16, P17, P18, P21, P22, P24, P25, P30, P31, P34, P37, P38, P39, P42, P43, P44, P45, P46, P49, P50, P51, P52, P54, P55, P56, P62, P63, P65, P66, P69, P70, P71, P72, P73]

Feature (d): Protecting the child from parental conflict

Description: strategies to protect the children from parents' litigating attitudes and behaviours

Source: [P2, P14, P21, P24, P38, P42, P59, P63, P69]

Feature (e): Maintaining a sense of stability

Description: children need to have their perception of stability protected throughout the dispute and after the case is closed

Source: [P2, P4, P7, P10, P11, P13, P15, P16, P17, P18, P21, P22, P23, P24, P25, P26, P30, P33, P40, P42, P43, P47, P48, P49, P52, P54, P55, P60, P63, P67, P69, P72]

Feature (f): Addressing the Children Act's welfare check-list

Description: the tool used by English legal actors to address the best interests of the child

Source: [P49, P50, P51, P52, P53, P54, P55, P56, P57, P58, P59, P65, P66, P70, P71, P72]

Candidate Theme 3: The Child's Role in the Decision-making process

General description: elements of the decision-making process regarding the child's role during a child custody dispute case

Feature(a): Passive Role

Description: impressions regarding the child's secondary role in the decision-making process

Source: [P1, P2, P7, P9, P10, P18, P19, P26, P31, P41, P49, P51, P55, P58, P69, P73]

Feature (b): "The child is heard only through a Psychosocial/CAFCASS Evaluation"

Description: perceptions that the child can only be heard when they are evaluated by a psychologist and/or social worker

Source: [P1, P2, P3, P5, P6, P9, P24, P27, P31 P34, P42, P44, P45, P50, P55, P65]

Feature (c): The children as subjects of rights and as an active actor of their reality

Description: legal actors' perceptions regarding the child's relevant role in the decision-making process

Source: [P9, P10, P13, P16, P18, P21, P24, P35, P38, P39, P42, P44, P49, P50, P51, P54, P53, P67, P73]

Feature (d): "There is no need to hear the child if there is a parental agreement"

Description: legal actors' perceptions that see young children speaking of wishes and views as valueless

Source: [P4, P35, P42, P44, P49]

Feature (e): Between inadequacy and lack of skills: "I do not hear the child"

Description: legal actors' perceptions that articulate their inability to properly hear the child and/or the inadequacy of doing so

Source: [P1, P2, P5, P7, P24, P27, P28, P42, P44, P46]

Feature (f): Child's perspective crucial for the decision-making

Description: legal actors' perception that sees young child's speaking of wishes and views as very important during the child custody dispute

Source: [P2, P4, P8, P9, P20, P24, P31, P35, P38, P45]

Feature (g): Listening to the Child's voice: the older, the better

Description: legal actors' perceptions that see the older child as easier informants of their wishes and views during the child custody dispute

Source: [P7, P24, P25, P27, P47, P49, P50, P51, P52, P53, P54, P55, P56, P57, P58, P64, P67, P69, P70, P71]

Candidate Theme 4: Psychologists' practice

**General description**: roles, characteristics, procedures and/or strategies that depict the psychologists' practice in child custody cases

Source: [P9, P12, P21, P35, P36, P38, P49, P61, P62, P64, P65, P67, P68, P69]

Candidate Theme 5: Social Workers' practice

**General description**: roles, characteristics, procedures and/or strategies that depict the social workers' practice in child custody cases

Source: [P13, P14, P24, P26, P63, P65, P70, P72, P73]

Candidate Theme 6: Independent experts' practice

**General description**: roles, characteristics, procedures and/or strategies that depict the independent experts' practice in child custody cases

Source: [P37, P40, P51, P59, P60, P63, P64, P65, P66]

Candidate Theme 7: Lawyers' Practice

**General description**: roles, characteristics, procedures and/or strategies that depict the lawyers' practice in child custody cases

Source: [P1, P3, P5, P15, P18, P24, P27, P29, P32, P33, P34, P42, P45, P47, P53, P54, P55, P56, P57, P58, P72]

Candidate Theme 8: Judges' Practice

**General description**: roles, characteristics, procedures and/or strategies that depict the judges' practice in child custody cases

Source: [P5, P16, P19, P27, P34, P35, P42, P46, P50]

Candidate Theme 9: Assessing BIC in Custody Cases: Context, Procedures and Professional Practice

**General description**: contextual and procedural issues regarding the assessment process carried out by psychosocial staff

Feature (a): 'Psychosocial Study': the Brazilian model

Description: characteristics of the 'psychosocial study' carried by Brazilian psychologists and social workers during the child custody dispute

Source: [P2, P3, P4, P8, P10, P12, P13, P14, P21, P22, P23, P24, P26, P31, P34, P35, P36, P39, P41, P42, P43]

Feature (b): 'Children and Family Court Advisory and Support Service – CAFCASS': the English model

Description: characteristics of the assessment carried by English social workers from CAFCASS during the child custody dispute

Source: [P49, P50, P51, P52, P53, P54, P56, P57, P59, P60, P67, P69]

Feature (c): Procedures, tools and practices to assess BIC

Description: legal actors' procedures, sources and tools to asses BIC in a custody decision-making scenario

(c.1)What is assessed?

(c.1.1) Child's Perspective [P13]

(c.1.2) Child's Development Stage and Specific Needs [P10, P12, P13, P17, P24, P44, P70]

(c.1.3) Child's Daily Life and Routine [P1, P3, P8, P11, P13, P24, P34, P39, P40 P44, P70]

(c.1.4) Family Dynamic and its Reality [P4, P5, P8, P12, P17, P24, P27, P42]

(c.1.5) Child-parent Relationship [P5, P8, P11, P13, P14, P15, P24, P33, P34, P35, P37, P38, P39, P43, P44, P45, P53, P64, P66, P72, P73]

(c.1.6) Parenthood & Co-parenting Skills [P3, P8, P10, P11, P13, P17, P20, P24, P35, P37, P43, P50, P64, P68, P69, P70, P72]

(c.1.7) Health Care [P1, P11, P13, P24, P42]

(c.1.8)Neglect, Maltreatment & Risk Factors [P6, P10, P44, P42, P56, P62, P63, P69]

(c.2) Sources of Information

(c.2.1) School & Carer and Protection Network [P1, P14, P15, P17, P20, P22, P24, P26, P28, P36, P37, P39, P40, P41, P42, P43, P44, P46, P47, P52, P68, P69, P70]

(c.3) Tools and Strategies to Assess BIC

(c.3.1) Interviewing Parents and Other Family Members [P8, P9, P12, P16, P24, P25, P26, P35, P36, P38, P39, P43, P44, P64, P67]

(c.3.2) Interviewing the Child [P8, P9, P12, P16, P24, P35, P37, P38, P41, P43, P66, P69, P71]

(c.3.3) Visiting the Family Home [P2, P9, P26, P39, P40, P41]

Candidate Theme 10: Decision-making Process

**General description**: characteristics related to the process of making a decision regarding child custody after parental separation

Feature (a): "There is no need to hear the child, if there is a parental agreement"
Description: legal actors' perceptions that see young child's speaking of wishes and views as needless during the child custody dispute
Source: [P4, P35, P42, P44, P49]

Feature (b): Between inadequacy and lack of skills: "I do not hear the child"
Description: legal actors' perceptions that inform their inability to properly hear the child and/or the inadequacy of doing it so
Source: [P1, P2, P5, P7, P24, P27, P28, P42, P44, P46]

Feature (c): Child's perspective crucial for the decision-making
Description: legal actors' perception that sees young child's speaking of wishes and views as very important during the child custody dispute
Source: [P2, P4, P8, P9, P20, P24, P31, P35, P38, P45]

Feature (d): Listening to the Child's voice: the older, the better

Description: legal actors' perceptions that see older child as easier informants of their wishes and views during the child custody dispute
Source: [P7, P24, P25, P27, P47, P49, P50, P51, P52, P53, P54, P55, P56, P57, P58, P64, P67, P69, P70, P71]

Feature (e) Trading-off interests
Description: legal actors' perceptions regarding of how to trade off child's needs during the child custody dispute
Source: [P10, P14, P25, P29, P42]

Feature (f): Address the child's interpersonal contexts
Description: legal actors' perceptions that see the child's interpersonal relationships and interactions as important inputs during the child custody dispute
Source: [P2, P13, P15, P18, P36, P44, P45, P49, P54, P55, P63]

Candidate Theme 11: Child Arrangements

**General description:** types of child arrangements after parental separation

Feature (a): Joint Custody

Description: legal actors' perceptions of joint custody

(a.1) Concept [P5, P44, P45]

(a.2) Misunderstanding joint custody [P6, P9, P15, P16, P22, P25, P31, P34, P43, P44]

(a.3) The perfect arrangement [P1, P3, P11, P18, P29, P42, P46]

(a.4) "It is settable regardless of the parental dynamic" [P1, P11, P16, P21]

(a.5) Conditional Joint Custody [P2, P4, P5, P6, P7, P15, P17, P19, P23, P24, P27, P29, P30, P31, P34, P35, P39, P44, P50, P63, P67, P73]

(a.6) "It is not to every family" [P4, P5, P6, P7, P8, P9, P12, P13, P14, P15, P17, P19, P20, P21, P23, P24, P26, P28, P30, P31, P35, P36, P39, P40, P41, P44, P57, P62, P63, P72, P73]

Feature (b): "The best arrangement is the one that fits the family best"

Description: legal actor's perceptions that understand the best arrangement is the one that fits the family's reality and possibilities

Source: [P6, P7, P8, P9, P10, P12, P15, P20, P22, P27, P36, P37, P38, P39, P41, P45, P49, P50, P51, P52, P53, P55, P57, P59, P62, P63, P64, P65, P66, P68, P69, P70, P71, P73]

Feature (c): Shared Caring

Description: legal actor's perceptions on shared care

Source: [P8, P15, P16, P17, P22, P24, P27, P38, P39, P43, P44, P45, P47, P48, P49, P65, P73]

Candidate Theme 12: Legal actors' biases and BIC

**General description**: legal actors' biases during the process of making a custodial decision

Feature (a): Gender

Description: legal actors' biases related to the parent's gender

Source: [P3, P4, P17, P28, P31, P42, P45, P47, P67]

Feature (b) Personal beliefs

Description: legal actors' biases related to their personal beliefs

Source: [P3, P7, P9, P10, P38, P47, P49, P50, P71]

Candidate Theme 13: (Mis)Understanding BIC

**General description**: elements of child custody decision-making that lead to misunderstanding and/or misuse of the BIC framework in order to justify conceptions, attitudes, ideas, and/or thoughts.

Feature (a): BIC as a rhetorical resources

Description: rhetorical use of 'the best interests of the child' to justify any action and/or argument within the dispute. Usually, this rhetorical use does not address BIC properly and/or does not focus on the child's needs

Source/Units of analysis: [P9, P11, P14, P18, P31, P32, P37, P43]

Feature (b): "It has nothing to do with Psychology"

Description: psychologists' ideas and/or conceptions that do not recognise BIC as part of their practice

Source: [P2, P8, P10, P35, P37]

Feature (c): Focusing on and addressing parents' interests instead

Description: attitudes and/or practices that use "BIC speech" to address and/or highlight adult's interests instead of the child's

Source: [P4, P5, P7, P8, P9, P10, P11, P15, P18 P33, P34, P35, P42, P43, P54, P65, P73]

Feature (d): "No 'child maintenance', no contact with the child"

Description: parents' perspectives that misunderstand BIC by making the contact between the child and the non-custodial parent conditional upon the making of maintenance payments

Source: [P2, P3, P5, P27, P29, P31, P45, P50]

Candidate Theme 14: Hindering BIC

General description: child custody scenario's elements regarding parenting issues towards the child during the custody dispute that can hinder the child's best interests.

Candidate Theme 15: Legal actors' Practice

General description: legal actors' practices during the decision-making process

Source: [P2, P7, P19, P42, P47, P48]
