# Supplementary material for: Making sense out of uncertainty: cognitive strategies in the child custody decision-making process
Source: Front Psychol. 2024 Jul 15;15:1387549. doi: 10.3389/fpsyg.2024.1387549 (PMC11284646; doi:10.3389/fpsyg.2024.1387549)
Supplement: Supplementary file 5 [file Table_5.pdf]

Supplementary Material 5

Article: Making sense out of uncertainty: cognitive strategies in child custody decision-making process

Journal: Frontiers in Psychology

Authors: Josimar Antônio de Alcântara Mendes; Thomas Ormerod

Anchoring per Country/Category of Legal Actor

Table 48 shows how each theme, feature and highlight are anchored in the data, taking into account each participant's ID. This ID is shown on Appendix H. As explained on the method section, the anchoring is just a tool used to provide the results' confirmability. **It should not be seen as a quantitative measure in which "the larger the number of supporters (participants) pointed, the more significant is the theme".**

Themes' Data Anchoring According to Each Participant

Theme	Participant ID[a]
Theme CS1: Promoting BIC in Child Custody Cases<br>(CS1.1) *Preserving basic (material-physiological) needs and rights*[1]<br>(CS1.2) *Enhancing the child's psychosocio-emotional well-being*[2]<br>(CS1.3) *Preserving the relationship with both parents*[3]<br>(CS1.4) *Protecting the child from parental conflict*[4]<br>(CS1.5) *Maintaining the sense of stability*[5]<br>(CS1.6) *Addressing the Children Act (1989)'s welfare check-list*[6]	1 – P2, P3, P4, P5, P6, P7, P8, P9 P11, P12, P13, P14, P16, P17, P22, P24, P26, P27, P29, P31, P32, P33, P34, P39, P42, P43, P44, P45, P46, P47, P48, P49, P50, P53, P55, P59, P64, P69, P70, P71, P73<br>2 – P2, P3, P4, P5, P6, P7, P8, P9, P11, P13, P14, P17, P20, P22, P26, P27, P29, P31, P33, P42, P43, P44, P45, P48, P49, P53, P54, P55, P56, P59, P63, P64, P65, P66, P70, P72, P73<br>3 – P1, P2, P3, P4, P7, P10, P11, P13, P16, P17, P18, P21, P22, P24, P25, P30, P31, P34, P37, P38, P39, P42, P43, P44, P45, P46, P49, P50, P51, P52, P54, P55, P56, P62, P63, P65, P66, P69, P70, P71, P72, P73

	4 – P2, P14, P21, P24, P38, P42, P59, P63, P69<br>5 – P2, P4, P7, P10, P11, P13, P15, P16, P17, P18, P21, P22, P23, P24, P25, P26, P30, P33, P40, P42, P43, P47, P48, P49, P52, P54, P55, P60, P63, P67, P69, P72<br>6 – P49, P50, P51, P52, P53, P54, P55, P56, P57, P58, P59, P65, P66, P70, P71, P72
Theme CS2: Decision-making Process<br>(CS2.1) *"There is no need to hear the child if there is a parental agreement"*[7]<br>(CS2.2) *Between inadequacy and lack of skills: "I do not hear the child"*[8]<br>(CS2.3) *Listening to the child's voice: the older, the better*[9]<br>(CS2.3.1) "It is easier to deal with": they can speak their minds[10]<br>(CS2.4) *Trading-off interests*[11]<br>(CS2.5) *Addressing the child's interpersonal contexts*[12]<br>(CS2.6) *The children as subjects of rights and as an active agent in their reality*[13]	7 – P4, P35, P42, P44, P49<br>8 – P1, P2, P5, P7, P24, P26, P27, P28, P31, P42, P44, P46<br>9 – P7, P24, P25, P27, P47, P49, P50, P51, P52, P53, P54, P55, P57, P58, P64, P67, P69, P70, P71<br>10 – P1, P3, P4, P5, P7, P8, P9, P15, P18, P29, P41, P44, P45, P46, P47, P50, P54, P55<br>11 – P10, P14, P25, P29, P42<br>12 – P2, P13, P15, P18, P36, P44, P45, P49, P54, P55, P63<br>13 – P2, P4, P8, P9, P10, P13, P16, P18, P20, P21, P24, P31, P35, P38, P39, P42, P45 P44, P49, P50, P51, P53, P67, P73
Theme CS3 : Assessing BIC in Child Custody Cases: Procedures, Sources and Tools<br>(CS3.1) *What is assessed?*<br>(CS3.1.1) Child's development stage and specific needs[14]<br>(CS3.1.2) Child's daily life and routine[15]<br>(CS3.1.3) Family dynamic and its reality[16]<br>(CS3.1.4) Child-parent relationship[17]<br>(CS3.1.5) Parenthood & Co-parenting skills[18]<br>(CS3.1.6) Health care[19]<br>(CS3.1.7) Neglect, Maltreatment & Risk factors[20]<br>(CS6.2) *Sources of information: school, caregivers and protection network*[21]<br>(CS6.3) *Tools and strategies to assess BIC*<br>(CS6.3.1) Interviewing parents and/or other family members[22]<br>(CS6.3.1.1) Aspects that are looked for during the interview[23]<br>(CS6.3.2) Interviewing the child[24]	14 – P10, P12, P13, P17, P24, P44, P70<br>15 – P1, P3, P8, P11, P13, P24, P34, P39, P40, P44, P70<br>16 – P4, P5, P8, P12, P17, P24, P27, P42<br>17 – P5, P8, P11, P13, P14, P15, P24, P33, P34, P35, P37, P38, P39, P43, P44, P45, P53, P64, P66, P72, P73<br>18 – P3, P8, P10, P11, P13, P17, P20, P24, P35, P37, P43, P50, P64, P68, P69, P70, P72<br>19 – P1, P11, P13, P24, P42<br>20 – P6, P10, P44, P42, P56, P62, P63, P69<br>21 – P1, P14, P15, P17, P20, P22, P24, P26, P28, P36, P37, P39, P40, P41, P42, P43, P44, P46, P47, P52, P68, P69, P70<br>22 – P8, P9, P12, P16, P21, P24, P25, P26, P35, P36, P38, P39, P43, P44, P63, P64, P65, P66, P67, P68, P70<br>23 – P12, P21, P24, P39, P68<br>24 – P8, P9, P12, P16, P24, P35, P37, P38, P41, P43, P66, P69, P71

(CS6.3.3) Visiting the family household[25]	25 – P2, P9, P24, P26, P39, P40, P41
Theme CS4 : Hindering BIC<br>(CS4.1) *Exclusion position*[26]	26 – P1, P2, P7, P9, P10, P18, P26, P49, P51, P58, P73
Theme CS5: Assessing BIC in Child Custody Cases: Evaluation Services<br>(CS5.1) *'Psychosocial Study': the Brazilian model*<br>(CS5.1.2) To intervene or not to intervene, that is the question[27]<br>(CS5.2) *'Children and Family Court Advisory and Support Service – CAFCASS': the English model*<br>(CS5.2.3) Risk-avoidance practice[28]	27 – P8, P13, P21, P22<br>28 – P56, P67
Theme CS6: Child Custody Arrangements<br>(CS6.1) *Joint Custody: between parental dynamics and conditions*<br>(CS6.1.1) "It is what the Law determines"[29]<br>(CS6.1.2) The perfect arrangement[30]<br>(CS6.1.3) "It is settable regardless of the parental dynamic"[31]<br>(CS6.1.4) Conditional joint custody: "It is not to every family"[32]<br>(CS6.2) *"The best arrangement is the one that fits the family best"*[33]	29 – P1, P3, P11, P18, P29, P42, P46<br>30 – P2, P3, P4, P5, P6, P11, P12, P15, P16, P18, P20, P21, P24, P28, P29, P34, P43, P44, P45, P46, P47<br>31 – P1, P11, P16, P21<br>32 – P2, P4, P5, P6, P7, P15, P17, P19, P23, P24, P27, P29, P30, P31, P34, P35, P39, P44, P50, P63, P67, P73<br>33 – P6, P7, P8, P9, P10, P12, P15, P20, P22, P27, P36, P37, P38, P39, P41, P45, P49, P50, P51, P52, P53, P55, P57, P59, P62, P63, P64, P65, P66, P68, P69, P70, P71, P73
Theme CS7 : Strategies to Avoid 'BIC-Harming Parental Litigation'<br>(CS7.1) *Self-arrangement: empowering the family*[34]<br>(CS7.2) *Educating parents*[35]<br>(CS7.3) *Mediation & Conciliation*[36]	34 – P2, P4, P5, P7, P13, P14, P20, P24, P27, P31, P34, P35, P42, P43, P44, P46, P49, P50, P51, P52, P53, P54, P55, P72, P73<br>35 – P1, P3, P5, P16, P25, P26, P27, P37, P39, P41, P42, P43, P46, P47, P49, P55, P57, P60, P66, P67, P70<br>36 – P16, P20, P28, P29, P31, P42, P43, P47, P50, P51, P53, P54, P56, P58, P59, P63, P66, P70, P72
Theme CS8: (Mis)Understanding BIC<br>(CS8.1) *Focusing on and addressing parents' interests instead of child's*[37]<br>(CS8.2) *"It has nothing to do with Psychology"*[38]<br>(CS8.3) *BIC as a rhetorical resource*[39]	37 – P2, P8, P10, P35, P37<br>38 – P4, P5, P7, P8, P9, P10, P11, P15, P33, P34, P35, P42, P43, P54, P65, P73<br>39 – P9, P11, P14, P18, P31, P32, P37, P43

Theme CS9: Dichotomies in Lawyers' Practice<br>(CS9.1) *Enrolling the dispute*[40]<br>(CS9.2) *Putting parents' interests first*[41]<br>(CS9.3) *Safeguarding the child's welfare*[42]<br>(CS9.4) *Seeing and addressing the child's best interests through the parents' interests*[43]	40 – P1, P3, P5, P15, P18, P27, P29, P45, P47, P54, P56, P58, P72<br>41 – P1, P18, P24, P29, P54, P57, P61<br>42 – P5, P27, P32, P33, P34, P55, P56, P58<br>43 – P34, P53, P54, P56, P57
Theme CS10: Legal actors' biases and BIC<br>(CS10.1) *Gender*[44]<br>(CS10.1.1) Misogyny[45]<br>(CS10.2) *Personal beliefs*[46]	44 – P3, P4, P5, P7, P17, P28, P31, P42, P45, P47, P67<br>45 – P5, P21, P47<br>46 – P3, P7, P9, P10, P38, P47, P49, P50, P71

[a] IDs according to information available in Online Source 2

According to below, data from 'psychologists', 'judges' and 'lawyers' categories were the most represented within the themes, with a total of 69% predominance. The same pattern was observed in relation to their representativeness within the number of participants, where they also presented as the top three categories.

Representativeness of Each Participant Category Within the Themes

**Representativeness Within the Themes**		
**Category**	**n**	**%**
Psychologists	243	25
Judges	214	22
Lawyers	214	22
Social Workers	171	18
Prosecutors	128	13
**TOTAL**	970	100
