# Supplementary material for: Making sense out of uncertainty: cognitive strategies in the child custody decision-making process
Source: Front Psychol. 2024 Jul 15;15:1387549. doi: 10.3389/fpsyg.2024.1387549 (PMC11284646; doi:10.3389/fpsyg.2024.1387549)
Supplement: Supplementary file 6 [file Table_6.pdf]

Supplementary Material 6

Article: Making sense out of uncertainty: cognitive strategies in child custody decision-making process

Journal: Frontiers in Psychology

Authors: Josimar Antônio de Alcântara Mendes; Thomas Ormerod

Reflexivity

I completed my bachelor's degree at the Catholic University of Brasília, Brazil. In the middle of the course, I enrolled in the module 'Conjugal and Family Psychology,' which introduced me to the Systemic approach and Family Therapy. I became deeply interested in 'Systemic Thinking' and its unique perspective on family dynamics. Subsequently, I actively participated in action-research projects focused on vulnerable families within the Systemic approach.

During my final year, I had a placement at the court of law in Brasília, Brazil, serving as an intern at the psychosocial service responsible for evaluating families and children involved in child custody cases post-parental separation. This service also embraced the Systemic approach, and I dedicated a year to this role. This experience proved decisive for my career, as it crystallized my aspiration to utilize the Systemic approach when working with families undergoing divorce and child custody disputes. I was so inspired that my final year dissertation delved into a systemic perspective on the best interests of the child in child custody cases.

I graduated in 2011 and commenced my master's program at the University of Brasília, Brazil, in the same year. My master's dissertation critically examined allegations of parental

alienation in child custody cases from a systemic perspective. When I defended my master's dissertation[1] in 2013, 'parental alienation' was a prominent and debated topic in Brazil. At that time, my work stood out as one of the few that proposed a critical approach, taking into consideration scientific, technical, clinical, and ethical issues through a systemic lens.

In 2014, I began teaching at a higher education institution, where I convened modules such as 'Forensic Psychology', 'Crisis Intervention', and 'Interventions in Situations of Risk'. Additionally, I supervised final year students counseling couples and families using 'Systemic Couple and Family Therapy'. Alongside teaching, I worked as a couples and family therapist under the Systemic approach. I maintained both roles until I relocated to the UK to commence my PhD in 2017.

My research interests have closely aligned with my professional journey. My publications have delved into various topics, including: a) the best interests of the child in child custody disputes; b) destructive divorce; c) parental alienation; d) the systemic view and legal actors; e) coping and rationalization in lawyers working in child custody cases; and f) a systemic bioecological view of child custody cases.

My professional journey, research pursuits, and published works have significantly influenced my perspective on family dynamics. I conceptualize the family as a living, dynamic entity that functions as a system. I contend that the relational interactions within a family are interdependent, intricate, and diverse, capable of yielding various outcomes—from enhancing family well-being to contributing to dysfunctionality, leading to inadaptation, disorganization, and psychological distress. This complexity underscores the potential for every family, particularly during challenging times, to self-improve and evolve. I firmly believe that each family harbors untapped potentialities, often undiscovered by both the family members themselves and the professionals supporting them.

---

[1] In Brazil, a master's degree typically takes at least two years to complete.

Parental separation, in this context, represents a moment of struggle and crisis for the family, shedding light on both its strengths and dysfunctional aspects. Therefore, I posit that the role of psychosocial professionals and the judiciary in such situations is to comprehend the family's struggle and facilitate their navigation through it without pathologizing, criminalizing, or hastily resorting to medication for the ‘post-divorce phenomena’.

Concerning the child or adolescent involved in child custody cases, I view them as active and integral participants within the family dynamic, contributing significantly to its intricacies. I advocate for recognizing the child or adolescent as a systemic protagonist in these cases, rather than a passive observer. While they do not make decisions, their ability to express their perspectives and thoughts deserves genuine consideration.

These conceptualizations of family, divorce, the roles of legal actors, and the judiciary’s functions permeated the entire research process for this study, with a particular emphasis on engaging in a ‘dialogue with the data’ and constructing themes and features.

It is crucial to highlight that, before undertaking my PhD studies, my prior research and professional experience led me to suspect that family development issues related to divorce and crises could impact the decision-making process in child custody cases. However, the specifics of how and why remained unclear. Through this study, I have gained insights into the contextual nature of these issues, understanding how they amplify the level of uncertainty, thereby intensifying the challenges in the decision-making process.
