# Supplementary material for: Making sense out of uncertainty: cognitive strategies in the child custody decision-making process
Source: Front Psychol. 2024 Jul 15;15:1387549. doi: 10.3389/fpsyg.2024.1387549 (PMC11284646; doi:10.3389/fpsyg.2024.1387549)
Supplement: Supplementary file 7 [file Table_7.pdf]

Supplementary Material 7

Article: Making sense out of uncertainty: cognitive strategies in child custody decision-making process

Journal: Frontiers in Psychology

Authors: Josimar Antônio de Alcântara Mendes; Thomas Ormerod

Themes, features and excerpts

Themes are presented according to a hierarchy of attributes: a) a *theme*: emerged from meaningful content in the dataset; b) *feature*: describes characteristics of the theme; and c) *highlight*: refers to relevant issues arising within a feature. Participants' quotations are linked to their ID, composed of their country ('BR'; 'EN') and category ('Jd' = Judge; 'Lw' = Lawyer; 'Pr' = Prosecutor; 'Psy' = psychologist; SW = Social Worker). Brazilian participants have their city pointed in their ID too (BsB = Brasília; POA = Porto Alegre; SP = São Paulo).

Table 2

Selection Heuristic Domain's Themes, Features and Highlights

THEME	FEATURES	DESCRIPTION	KEY EXCERPTS
**Theme CS1: Promoting BIC in Child Custody Cases**	(CS1.1) *Preserving basic (material-physiological) needs and rights*	Feature CS1.1 captures elements that legal professionals select in child custody cases to promote the child's basic needs and rights	"[I look over] all the aspects that refer to the basic needs […] housing, physical well-being, clothing, food." **BR_BsB.SW.02**<br>"Physically safe environment that kind of applies for their educational welfare, it will keep them healthy, give them food and clothes to wear." **EN_Psy.06**
	(CS1.2) *Enhancing the child's psychosocio-emotional well-being*	Feature CS1.2 captures elements that legal professionals select in child custody cases to address the child's psychosocio-emotional well-being	"[One has to look after children's] mental health, preserving their emotional and social well-being." **BR_BsB.Lw.02**<br>"Regarding post-separation, the emotional impact on a child is something that is a real concern." **EN_Lw.02**
	(CS1.3) *Preserving the relationship with both parents*	Feature CS1.3 captures elements that legal professionals select in child custody cases that are related to the child's relationship with the non-custodial parent	"[to protect] this coexistence with the other parent or even with other people of the family is important and is a child's right." **BR_Pr.03**<br>"I think you have to try very hard to make sure that you keep the relationship going between the child and both parents." **EN_Jd.01**
	(CS1.4) *Protecting the child from parental conflict*	Feature CS1.4 captures elements that legal professionals select in child custody cases related to protecting the child from the litigious dispute	"I think in such cases [child custody] the peace of the child is jeopardised because the child is involved in the dispute, what is taken from her/him is precisely this, it is peace and tranquillity." **BR_POA.Psy.02**<br>"First thing is to form a clear agreement that could enable the parents to agree on a certain course of action, which will not put the child in a conflict situation. So, sparing the child from that conflict by having a clear agreement." **EN_SW.01**

		between the parents	
	(CS1.5) *Maintaining a sense of stability*	Feature CS1.5 captures elements that legal professionals select in child custody cases that are related to the child's perception of continuity	"What most affects [children] in a divorce situation, is the loss of the current stability." **BR_BsB.Lw.03**<br>"I think that the thing is that kind of stability is the background, the bedrock of the child's life and you don't disturb it. They need their interests and they need stability outside of the parental relationship." **EN_SW.01**
**Theme CS2: Decision-making Process**	(CS2.5) *Addressing the child's interpersonal contexts*	Feature CS2.5 captures legal professionals' perceptions that the child's interpersonal relationships and interactions are important inputs for the custodial decision-making process	"The best interests of the child go, necessarily, through the family's well-being." **BR_BsB.Jd.02**<br>"The best interests of the child cannot be seen in an isolated way, it has to be seen related to a context and to the family's capabilities and/or to the environment in which the child is in." **BR_BsB.SW.01**<br>"So it is important that [children] have a relationship with the extended family, because that's their roots, that's their connection, they have to." **EN_Lw.02**
**Theme CS3: Assessing BIC in Child Custody Cases: Procedures, Sources and Tools**	(CS3.1) *What is assessed?*<br>Feature CS3.1 captures aspects related to the child's development, routine, emotional bonds, and characteristics of the family context that are assessed during the evaluation process	(CS3.1.1) Child's development stage and specific needs	"I think each stage [of development] is specific, right? I mean, the very condition of being a child, a younger child, an older child, means different interests and rights." **BR_BsB.SW.01**<br>"We try to figure out the child's needs in each step of their development and what's the parent's role in this whole process. Based on this information, we can be more confident when suggesting sole physical or joint custody." **BR_BsB.SW.01**
		(CS3.1.2) Child's daily life and routine	"I examine which one of them [the parents], within their routine, better accommodates the child, who has more availability of time, which is more able to support the child on a daily basis; for example, schooling, help with homework." **BR_BsB.Jd.01**<br>"What kind of things did they do? It's not just about all activities and doing nice things, because you also have to do things in the home, like living normal home life, which is not just about having fun all the time. There is cooking, having a meal together, sitting at the table, watching a TV program together." **EN_SW.02**

**Theme CS3: Assessing BIC in Child Custody Cases: Procedures, Sources and Tools**	(CS3.1) *What is assessed?*<br><br>Feature CS3.1 captures aspects related to the child's development, routine, emotional bonds, and characteristics of the family context that are assessed during the evaluation process	(CS3.1.3) The family dynamic and its reality	"[it is important] to analyse, to consider the family; what their morals are, their customs, how they have developed as a family." **BR_BsB.Lw.01**<br>"The psychosocial evaluation can bring out the panorama of family relations and the dynamics of how that family and the conflict work." **BR_Pr.01**
		(CS3.1.4) Child-parent relationship	"The relationship between them [parents and child] is evaluated, the level of complicity, the relationship they have […] the companionship, intimacy, whether they are emotionally close." **BR_BsB.Lw.01**<br>"Watching the parent play with the child is quite interesting, you know. Do they have to win all the time? What does that tell me about their relationship with the child? You know, how coercive are the parents in the way they talk to their child? Are they seeking to get the child as an ally, you know?" **EN_SW.04**
		(CS3.1.5) Parenthood & Co-parenting skills	"It is important to see which of them is more flexible in regard to maintaining the contact between the child and the other parent." **BR_BsB.SW.01**<br>"You look at the parenting, the parents' relationship styles and you look at whether or not they're sensitive to the needs of the child or whether they're unresponsive so they avoid or neglect the needs of the child and prioritize their own or whether they try to control the child within their relationship." **EN_SW.04**
		(CS3.1.6) Healthcare	"Sometimes, I ask one of the parents, 'Did the kid take all the needed vaccines for their age?' and if they answer, "Oh, I do not know because the vaccination card is with their mother"... well, the vaccination card is with the mother, but we still are talking about their kid, so they should have known." **BR_POA.SW.01**
		(CS3.1.7) Neglect, maltreatment & risk factors	"We tend to see risk and protection factors, not only in cases of vulnerability, even in a context of social vulnerability, even in contexts of families more privileged economically, we try to investigate what the emotional risk factors are, the ones for coexistence." **BR_BsB.Psy.03**<br>"The stimulation they receive, whether there's any neglect, whether there's a risk of chaotic living or whether there is any risk to the child posed by the parents because they have mental health problems, drug abuse and so forth." **EN_Psy.04**

	(CS3.3) *Tools and strategies to assess*<br>Feature CS3.3 captures tools and strategies that psychosocial staff use to assess the child's best interests in child custody cases	(CS3.3.1) Interviewing parents and/or other family members	"Interviews are conducted individually with each parent and sometimes with other relatives to help us understand how the family are organised and how their dynamic as a family is." **BR_POA.SW.01**<br>"I begin by interviewing both parents. So that is, that is the critical first step for me, is to give a picture of what's going on basically in the broadest sense possible." **EN_Psy.05**

Table 3

Evaluation Heuristic Domain's Themes, Features and Highlights

THEME	FEATURES	DESCRIPTION	KEY EXCERPTS
**CS4: Hindering the Best Interests of the Child**	(CS4.5) *Exclusion position*	Feature CS4.5 captures legal professionals' perceptions regarding the child's secondary role in the custodial decision-making process	"I do not see [the child] as an actor in this process." **BR_BsB.Jd.01**<br>"I do not see any active participation [of the child] in order to help us make a decision, no." **BR_BsB.Jd.02**<br>"What is terrible is that the child almost gets ignored as a person in the dispute between the parents." **EN_Lw.06**<br>"Ideally, the child should not know that there are court proceedings." **EN_Jd.03**
**Theme CS2: Decision-making Process**	(CS2.1) *"There is no need to hear the child if there is a parental agreement"*	Feature CS2.1 captures legal professionals' perceptions that a young child speaking of wishes, feelings and views is not needed during the custodial decision-making process, if there is an	"In the vast majority of cases where parents come to court and quickly reach an agreement, it would be rare for the child to be seen by the judge." **EN_Jd.01**<br>"That is, when the adults agree, we do not ask what the best interests of the child are. It is assumed that if the adults are on the same page, BIC is preserved, from this perspective." **BR_SP.Psy.01**

		agreement between the parents	
**Theme CS2: Decision-making Process**	(CS2.2) *Between inadequacy and lack of skills: "I do not hear the child"*	Feature CS2.2 captures legal professionals' inability to properly hear the child, or the inadequacy of doing so during the custodial decision-making process	"We rarely talk to the children […] The judge, by the number of cases, does not have the time to do this and he/she is not prepared to do it, he/she has no training to do this." **BR_SP.Jd.02**<br>"I do not like it [hearing the child] because when the child arrives to be heard by the judge, it is usually in the context of a conflict of loyalty." **BR_Pr.01**
	(CS2.3) *Listening to the Child's voice: the older, the better*	Feature CS2.3 captures legal professionals' perceptions that see the older child as a better informant of their wishes, feelings and views	"I confess that I prefer to talk with adolescents than with young children because there are no instruments and methodologies, and no specific training is needed for this." **BR_POA.SW.02**<br>"Especially if it's a child over age 8, 9, 10, when they are able to express their views. It's very difficult when they are very small, at 1, 2, 3, 4, the decision is made purely then from an objective perspective and maybe an expert might say as to what is in their best interest." **EN_Lw.03**<br>"It will depend on the age of the child. Obviously, that's one big parameter. It'll depend on whether the child has Gillick competency." **EN_Psy.05**
	(CS2.4) *Trading-off interests*	Feature CS2.4 captures legal professionals' perceptions regarding the ways in which they trade-off the various factors relating to the child's needs during the custodial decision-making process	"This [emotional bond] is a tie-breaker criterion. It's pointless to let the child with a parent that has a good income, lives nearby the school, is well-educated but does not have a good affective bonding with the child be granted custody, so affective issues will prevail." **BR_SP.Jd.03**<br>"In child custody cases, you have to weigh what is the best interests of that child concerning the type of custody, the coexistence arrangement, alimony, etc…" **BR_Pr.01**

	(CS2.6) *The children as subjects of rights and as active agents in their reality*	Feature CS2.6 captures the legal actor's perceptions regarding the relevance of the child's role in the custodial decision-making process	"I think there is the issue of the child being seen as a subject, as someone who feels, that can participate, who has an opinion and understands what is happening […] I think it [the child's role] has to be an active role, [they are] a protagonist for me, I think the child has to speak." **BR_BsB.Psy.02**<br>"[Children] cannot be seen as if they were an object, they cannot be objectified in the custody dispute […] as if one could do whatever one wants, as if the child were a clay mass that one can shape in whatever way one wants." **BR_BsB.Psy.03**
**Theme CS3: Assessing BIC in Child Custody Cases: Procedures, Sources and Tools**	(CS3.3) *Tools and strategies to assess BIC*<br><br>Feature (CS3.3) captures tools and strategies that psychosocial staff use to assess the child's best interests in child custody cases after parental separation	(CS3.3.2) Interviewing the child	"[When] I'm with the child or the young person, I try just to talk about their relationships generally, I usually am seeing them in school. So I ask them first about the school relationships just to kind of ease them into the discussion." **EN_Psy.07**<br>"I suggest the drawing [to start the interview], I can begin by observing how they decide what they are going to draw, how the negotiation is made." **BR_SP.Psy.01**
		(CS3.3.3) Visiting the family household	"Visit the place where the child lives and there you see the child in context, how he or she deals with that context of preference." **BR_BsB.Psy.02**<br>"I think that visiting the house is the most important instrument. Through the visit to the family household, it is possible to identify which space the child occupies in that context." **BR_SP.SW.02**

Table 4

Degrees of Freedom Heuristic Domain's Themes, Features and Highlights

THEME	FEATURES	DESCRIPTION	KEY EXCERPTS
**Theme CS1: Promoting BIC in Child Custody Cases**	(CS1.6) *Addressing the Children Act's welfare checklist*	Feature CS1.6 captures elements that English legal professionals consider in child custody cases in order to promote the child's welfare	"Well, I think the welfare checklist in Section 1, subsection 3 sets out all that you need to know [to address BIC]." **EN_Jd.01**<br>"Perhaps in Britain, the best interests of the child are represented by the welfare checklist […] So, for me, I always refer to the welfare checklist." **EN_SW.03**<br>"I tend, certainly, on a difficult case, to go through each element of the welfare checklist quite slavishly." **EN_Jd.01**
**Theme CS6: Child Custody Arrangements**	(CS6.1) *Joint Custody: between parental dynamics and conditions*<br><br>Feature CS6.1 captures legal professionals' perceptions regarding their understanding and application of the 'joint custody arrangement'	(CS6.1.1) "It is what the law determines"	"Today, the law determines that the legal custody is joint custody. Moreover, the law states that the rule is joint custody" **BR_BsB.Jd.02**<br>"The custody, as a rule, should be joint custody. I try to follow it because that is the rule, that is [what was] enacted" **BR_BsB.Jd.03**<br>"The law is clear, without agreement between the parties, the custody will be joint, except in rare hypotheses" **BR_POA.Jd.01**
	(CS6.2) *"The best arrangement is the one that fits the family best"*	Feature CS6.2 captures legal professionals' perceptions that the best arrangement is the one that best fits the family's reality and possibilities	"The best arrangement is the one which best fits the family. It is important to consider the characteristics of both child and family." **BR_SP.SW.01**<br>"Well, my thought is that you cannot have a one-size-fits-all policy. Every case is different. You've got a different dynamic between the child and the parents. And I think it's very dangerous to have very specific views about what is going to be best. I think you have to look at each case individually." **EN_Jd.04**<br>"Everything I have seen over the years since then has been a softening of that; along the lines of: it's whatever works for the family and it's whatever works for the child."

		**EN_SW.01**
(CS6.3) *Shared caring: not good enough to be applied but not too bad if the child has already adapted*	Feature CS6.3 captures the legal professionals' perceptions that understand the shared caring as a good option if it fits the family and the child's reality and needs	"Many parents already come up with this arrangement in court, it is no problem at all for me if it is working, that is what we want, what we are looking for. If the child has adjusted, it is perfect! However, if it will impose this routine on the child, I do not grant such an arrangement." **BR_BsB.Jd.01**<br>"That's not an arrangement that I personally like, I have reservations about that; but, if two parents come to me and say "this is what we agreed that should happen", then I would let it happen. But I think it's problematic." **EN_Jd.01**

Table 5

Outsourcing Decisions & Resolution Heuristic Domain's Themes and Features

THEME	FEATURES	DESCRIPTION	KEY EXCERPTS
**Theme CS3: Assessing BIC in Child Custody Cases: Procedures, Sources and Tools**	(CS3.2) *Sources of information: school, caregivers and protection network*	Feature CS3.2 captures inputs from school, caregivers or social services, taken into account during the evaluation	"The school is a great indicator, school performance and the child's behaviour at school is an indicator of the child's needs, the problems they are facing, and how these problems are presenting themselves." **BR_Pr.01**<br>"Input from the child's social network is an important reference point for the judge to be able to make that decision." **BR_Pr.01**<br>"Always talk to professionals, if possible, who knew the child... usually that would be a school teacher or the head of the school, who would give us some sort of insight into how the parents' dispute, because there's always a dispute involved, how it [parental dispute] is affecting the child." **EN_SW.01**

**Theme CS7: Strategies to Avoid ‘ BIC-Harming Parental Litigation’**	(CS7.1) *Self-arrangement: empowering the family*	Feature CS7.1 captures issues that advocate for self-arrangements for child custody by prioritising the family's capacity and competency to know and understand its own reality and needs.	"We have to try to give back their ability to solve their own issues. '*Oh, it is in your hands,*' we say, '*we will inform the judge, but the power* [to find a better solution] *is in your hands*'. Everything goes through the parents." **BR_BsB.SW.02**<br>"Many times an imposed solution, in the family court, can create and even increase the conflict, depending on the case." **BR_Pr.02**<br>"Parents come in all shapes and sizes, some might be wonderful, others might be not so wonderful, but at the end of the day, this is their child and the state and the court should not be intervenient unless they really have to." **EN_Jd.02**<br>"The court is not going to be there forever, the court isn't going to be involved in their lives in every decision, so if upon separation we can get them to work together and to come up with a plan together, then that kind of works for the future of the child." **EN_Lw.01**
**Theme CS7: Strategies to Avoid ‘ BIC-Harming Parental Litigation’**	(CS7.3) *Mediation & Conciliation*	Feature CS7.3 captures extra-judicial initiatives to promote family mediation or conciliation in child custody cases, intending to mitigate parental litigation and BIC hindering	"Mediation, conciliation… these things that can solve the situation without having to judicialize the issue." **BR_BsB.Psy.02**<br>"First, you will try family mediation, through dialogue, make the parties talk with the help of a third party, and get them to work out a solution." **BR_POA.Jd.02**<br>"I have been using parental mediation to try to help the parents separate the conjugal issues from parenting and it has helped a lot." **BR_SP.Jd.03**<br>"So, mediation is an opportunity for parents to try to solve their problems together. Without somebody, a stranger or a judge, for instance, having to make decisions on their behalf." **EN_Lw.04**<br>"Under the English system, parents are encouraged to go to meetings, mediation before going to court, but, again, there was a problem with funding mediation – it doesn't happen as often as it should." **EN_Jd.03**

Table 6

Custodial Arrangements Domain's Themes, Features and Highlights

THEME	FEATURES	DESCRIPTION	KEY EXCERPTS
**Theme CS6: Child Custody Arrangements**	(CS6.1) *Joint custody: between parental dynamics and conditions*<br><br>Feature CS6.1 captures legal professionals' perceptions regarding their understanding and application of the 'joint custody arrangement'.	(CS6.1.2) The perfect arrangement	"I rarely order a sole physical custody. Joint custody, to me, really, was an incredible breakthrough in the child protection issue" **BR_BsB.Jd.01**<br>"I understand that joint custody is the one that best meets it [BIC], precisely because it offers a balance of power in the exercise of family power" **BR_BsB.Jd.01**
		(CS6.1.3) "It is settable regardless of the parental dynamic"	"[the legislator wanted to say:] *It does not matter if you do not get along... the custody is joint, make it work, you will have to figure it out*". It puts they [parents] both on an equal footing. I think that is the most important thing" **BR_POA.Psy.02**
		(CS6.1.4) Conditional joint custody: "It is not for every family"	"As long as there is respect between parents; they do not need to be friends, but they need to be able to talk to each other with respect, talk and solve their children's stuff. […] If they fight, forget about it [granting joint custody]." **BR_BsB.Jd.02**<br>"I think if you have eliminated any particular risks for the child then I think joint custody is always going to be the best outcome, for the best interests of the child. Because children thrive the more people they have involved." **EN_Psy.08**

Table 7

Professional Practices Domain's Themes, Features and Highlights

THEME	FEATURES	DESCRIPTION	KEY EXCERPTS
**Theme CS8: (Mis)Understanding BIC**	(CS8.2) *"It has nothing to do with psychology"*	Feature CS8.2 captures how some psychologists do not see BIC as linked to their practice	"I think that within psychology no one discusses this [BIC]." **BR_BsB.Psy.03**<br>"Actually, we do not use that term [BIC] much. It's a legal term." **BR_BsB.Psy.01**<br>"I did not hear about the best interests of the child in psychology, this question is not asked." **BR_SP.Psy.03**
**Theme CS9: Dichotomies in Lawyers' Practice**	(CS9.1) *Enrolling the dispute*	Feature CS9.1 captures the lawyers' involvement in the parental litigation by enrolling themselves within, or increasing the acrimony between litigating parents	"It's very easy as lawyers to become aggressive, to become overly involved in a case to the point where all you are doing is being a mouthpiece for your client." **EN_Lw.04**<br>"There are times that lawyers do not help because they have those interests, interests in continuing the litigation, they want the fight because then they will have some financial benefit." **BR_SP.Jd.01**<br>"Sometimes you see that the lawyer is fighting more than the clients. If I take out the lawyers and leave the two parties here, they will calmly talk to each other...." **BR_SP.Jd.01**
	(CS9.2) *Putting parents' interests first*	Feature CS9.2 captures lawyers' practices and perspectives that put parents' interests before the child's	"The lawyer acts in the interest of their client, and often the interest of their client is not exactly in the best interests of the child. […] Sometimes, by defending the client's interest, the lawyer ends up violating the child's." **BR_BsB.Jd.01**<br>"My role is not to promote what is in the best interest of the child, my role is to advise my clients as to whatever their subjective opinion is, how would it be received by the law?" **EN_Lw.02**

**Theme CS9: Dichotomies in Lawyers' Practice**	(CS9.3) *Safeguarding the child's welfare*	Feature CS9.3 captures lawyers' practices and perspectives around commitments to safeguard the child	"The role of the lawyer in the context of the best interests of the child is to make the parents understand that the custody dispute does not concern them; it concerns the child." **BR_BsB.Lw.01**<br>"I got to keep bringing them back down to the basics: *this is about the child and it is a child-centred, child-focused decision that the courts make; it's not about what you think, it's not about what the other party thinks, this is about what is in this child's best interests.*" **EN_Lw.03**
**Theme 10: Legal professionals' biases and BIC**	(CS10.1) *Gender*	Feature CS10.1 captures the legal professionals' gender biases during the custodial decision-making process	"I think there is a gender issue there. It is crucial, because of biological factors and not because of cultural factors, that the mother breastfeeds, and I cannot impede a child to have breastfeeding." **BR_BsB.Jd.04**<br>"A baby will not be able to express his or her will, and of course the baby will want to go with the mother. Usually, the biggest bond for a baby is with the mother." **BR_POA.Lw.01**<br>"The tendency still, in Brazil, and that I can say, can be a gender problem. The child will stay with the mother. I would say, in Brazil, that if the mother wants to stay with her son, she hardly will not." **BR_Pr.06**<br>"In practice in England, the biggest problem we have really is that the child is nearly always placed with the mother. Even when you know there are drug difficulties, alcohol problems, it's very rare for the father to get custody of a child." **EN_Psy.08**<br>"Women end up being villainised in the family courts with this idea of parental alienation as being a practice more associated with women" **BR_POA.Psy.02**
	(CS10.2) *Personal beliefs*	Feature CS10.2 captures legal professionals' biases related to their personal beliefs that can affect BIC	"I feel that some decisions focus a lot on the personal values of the judge or the prosecutor." **BR_BsB.Psy.03**<br>"I have seen a judge talking about the bible in a judgment." **BR_BsB.Jd.03**<br>"Usually, the arguments are based on personal experiences, so the judge says: 'but my son is like this or like that' – they use themselves as a reference which can be good or bad [for the decision-making process]." **BR_SP.Lw.04**<br>"It is preferable to have a marriage that's not very good than a bad separation. So, if it is terrible to be separated, stay married." **BR_Pr.06**

**Theme CS7: Strategies to Avoid ' BIC-Harming Parental Litigation'**	(CS7.2) *Educating parents*	Feature CS7.2 captures the need to orientate and educate parents going through a custody dispute	"And also in the process of raising awareness of parenthood, responsible parenthood, [there are] parenting workshops in which parents are invited to talk about it, to discuss it. So, it is an issue of orientation, we have been seeking to guide parents." **BR_Pr.02**<br>"We try to guide the parent on how to have a less aggressive dialogue. Some techniques that we try to teach to these parts [parents]." **BR_Pr.02**<br>"There is something we always say to clients: you must not talk in a derogative manner about the other parent in front of the child, that is against their best interest." **EN_Lw.03**<br>"[We try to] educate them [parents] really about the effects on the child of acrimonious dispute." **EN_Psy.07**
**Theme CS5: Assessing BIC in Child Custody Cases: Evaluation Services**	(CS5.1) *Psychosocial study': the Brazilian model*	(CS5.1.2) To intervene or not to intervene, that is the question	[the psychosocial professionals' role] is to promote reflection, and intervention in some cases, where we perceive cases of vulnerability or risks that are spotted and referred to the support network." **BR_BsB.SW.02**<br>"When they [parents] come for an evaluation, they come very much in a position of defence. Therefore, I think it is a bit of an illusion for us to think that there will be an intervention, a big intervention. We can suggest interventions, of course, but our role here is evaluating." **BR_POA.Psy.02**
	(CS5.2) '*Children and Family Court Advisory and Support Service – CAFCASS': the English model*	(CS5.2.3) Risk-avoidance practice	"As with all cases of professionals working with risk, they [CAFCASS] are on the side of caution. Because ultimately, it's about protecting their own judgment. And covering themselves." **EN_Psy.08**<br>"I do think that they are a very risk-averse organization. They certainly have become that. So, for instance, they will always take the safest route, safest route even if it means that a child potentially might suffer by not having a relationship." **EN_Lw.04**

Table 8

BIC Speech Domain's Themes and Features

THEME	FEATURES	DESCRIPTION	KEY EXCERPTS
**Theme CS8: (Mis)Understanding BIC**	(CS8.1) *Focusing and addressing parents' interests instead of BIC*	Feature CS8.1 captures the misuse of BIC in support of, primarily, adults' needs or interests, instead of those of the child	"Many times [BIC] is confused with the parents' best interests." **BR_Pr.02**<br>"I understand that the best interests of the child are, actually, what would suit the best interests of the adults." **BR_SP.Psy.01**<br>"You don't say that. But, some parents are motivated by money. Money is really important to determine what is in the best interests of your child. Trust me, money!" **EN_Lw.02**
	(CS8.3) *BIC as a rhetorical resource*	Feature CS8.3 captures the use of BIC by legal professionals to justify any action and/or argument, even when it does not focus on the child's interests	"It seems to me that this expression [the 'best interests'] is utilised more as a figure of speech than to express thorough preoccupation. It sounds good when you say 'best interests of the child'. (…) It seems that the problem would be solved just by mentioning it [BIC]." **BR_SP.Psy.03**<br>"Since it does not seem to be something 'palpable', one can justify anything at the beginning, even to deny or to defend something, not to listen to the child or to listen to the child, to grant custody or to not grant a custody." **BR_POA.Lw.02**
**Theme CS9: Dichotomies in Lawyers' Practice**	(CS9.4) *Seeing and addressing the child's best interests through the parents' interests*	Feature CS9.4 captures lawyers' practices and perspectives that try to address the child's interests by articulating them via the parent's interests	"I will defend the child's best interests under the viewpoint of my client." **BR_SP.Lw.02**<br>"I try to achieve the outcome that my client wants, which should be linked back to what is the child's best interests. […] But, ultimately, it is not for me to determine what's in the child's best interests, it's for the client to determine with my advice and then we could forward their position." **EN_Lw.01**
